# Hypoxic Postconditioning Offers Neuroprotection Against Transient Cerebral Ischemia via Down‐Regulation of rno_piR_011022

**DOI:** 10.1111/cns.70295

**Published:** 2025-02-25

**Authors:** Meiyan Chen, Shanshan Duan, Guorong Chai, Lixuan Zhan, Linhui Peng, Weiwen Sun, En Xu

**Affiliations:** ^1^ Department of Neurology, Institute of Neuroscience, Key Laboratory of Neurogenetics and Channelopathies of Guangdong Province and the Ministry of Education of China, the Second Affiliated Hospital Guangzhou Medical University Guangzhou China

**Keywords:** apoptosis, cerebral ischemia, NR2B‐PSD95, rno_piR_011022, synaptic plasticity

## Abstract

**Background:**

Piwi‐interacting RNAs (piRNAs) are differentially expressed after cerebral ischemia. However, little is known about their roles in transient global cerebral ischemia (tGCI). Herein, we aim to elucidate the roles and the underlying molecular mechanisms of piRNAs in tGCI and cerebral ischemic tolerance induced by hypoxic postconditioning (HPC).

**Methods:**

The male rat models of tGCI and HPC were established in vivo. Oxygen–glucose deprivation/reoxygenation (OGD/R) was developed from primary hippocampal neurons in vitro. RNA‐sequencing, fluorescence in situ hybridization, and quantitative real‐time PCR were used for detecting piRNA expression. Immunohistochemistry, TUNEL staining, CCK8 assay, etc., were used to evaluate neuronal damage. Western blot was used to measure protein levels of NR2B, PSD95, and cleaved‐caspase 3.

**Results:**

The expression profiles of piRNAs in CA1 were significantly changed after tGCI. HPC downregulated the expression of the top 5 piRNAs associated with synaptic function. Notably, the knockdown of rno_piR_011022 not only alleviated neuronal apoptosis and enhanced synaptic plasticity after tGCI and OGD/R but also reduced methyl‐D‐aspartate (NMDA) receptor 2B (NR2B) expression and inhibited NR2B‐postsynaptic density 95 (PSD95) interaction following tGCI. HPC enhanced these inhibitory effects.

**Conclusion:**

This innovative study indicated that the down‐regulation of rno_piR_011022 plays an important role in HPC‐mediated neuroprotection against tGCI through inhibiting the NR2B‐PSD95 interaction.

## Graphical abstract

The expression profiles of piRNAs in CA1 were significantly changed after tGCI. HPC down‐regulated the expressions of the top 5 piRNAs associated with synaptic function, including rno_piR_000618, rno_piR_009428, rno_piR_017990, rno_piR_014971, and rno_piR_011022 induced by tGCI. Down‐regulation of rno_piR_011022 mediated by HPC enhanced synaptic plasticity through inhibiting the NR2B‐PSD95 interaction, thus ameliorating ischemic neuronal injury and improving the learning and memory functions of rats.

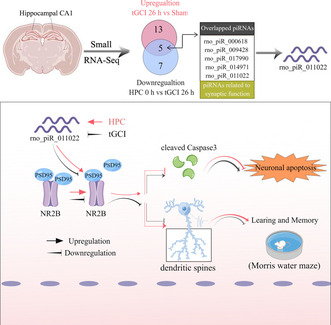

## 1 Introduction

Non‐coding RNAs (ncRNAs) are involved in the pathogenesis of acute cerebral ischemia [1, 2]. Whereas piwi‐interacting RNAs (piRNAs) have received less attention despite their abundant expression in the brain [3]. As a kind of small ncRNAs, mostly 24–32 nucleotides in length, they were first discovered in the *D. melanogaster* germline as a novel class of ‘long siRNAs’ [4]. More recently, piRNAs have attracted great interest because of their roles in maintaining cellular homeostasis. They are not only critical to transposon silencing in the germline, but also in somatic cells, including neurons [5, 6]. Dharap et al. first profiled 39,727 piRNAs in the cerebral cortex of adult rats subjected to transient focal ischemia. They found that many piRNAs are expressed in the brain and several are highly stroke‐responsive [7]. Similarly, we reported that a total of 5574 piRNAs were differentially expressed in hippocampal CA1 neurons at 26 h of reperfusion after transient global cerebral ischemia (tGCI) [8]. However, the functions and molecular mechanisms of piRNAs in cerebral ischemia are not fully understood.

Recent studies have shown that piRNAs are involved in cardiac remodeling [9], brain development, axonal regeneration, and memory formation [10, 11, 12]. Rajasethupathy et al. demonstrated that several piRNAs selectively enrich in neurons of Aplysia. The knockdown of PIWI or a specific piRNA, such as piR‐F, was found to impair long‐term facilitation [12]. These studies suggest that the roles of neuronal piRNAs are associated with learning and memory.

Recently we reported that synaptic and dendritic structures in CA1 were disturbed and the spatial learning and memory of rats were significantly impaired after tGCI. Conversely, hypoxic postconditioning (HPC) with 8% O_2_ for 2 at 24 h after ischemia reduces the expression of Piwil2 to protect CA1 neurons by restoring the structural integrity of dendritic spines, which subsequently improves the cognitive function of rats [8]. However, which piRNAs are the central regulators in the synaptic integrity of neurons and cognition of rats in tGCI and HPC‐induced ischemic tolerance is still unknown.

*N*‐methyl‐D‐aspartate receptor (NMDAR) is a heteromeric, ligand‐gated ion channel composed of seven subunits: NR1, NR2A‐D, and NR3A‐B^13^. Among them, NR2B is responsible for neurotransmission, neuronal plasticity, and is involved in learning and memory in the hippocampus [13, 14, 15]. Under ischemic conditions, glutamate would be excessively released into the synaptic cleft, activating NR2B [16]. Our previous study has demonstrated that tGCI increased extracellular glutamate release in CA1 [17]. Opened NMDAR‐coupled channels allow calcium ions to enter the intracellular compartment and activate calcium‐dependent enzymes, thus leading to apoptosis in ischemic human cardiomyocytes [18]. Moreover, NR2B modulates glutamate transmission and maintains excitatory synapse balance through interaction with postsynaptic density 95 (PSD95) [19, 20, 21]. In a forebrain ischemia model of rats, NR2B‐PSD95 interaction was markedly enhanced [22]. However, Tat‐NR2B9c could selectively reduce NR2B‐PSD95 interaction to protect against renal or cerebral ischemia [23, 24]. Nevertheless, it remains uninvestigated whether piRNAs play a crucial role in HPC‐induced neuroprotection against tCGI through regulating NR2B‐PSD95 interaction.

Herein, we aimed to illustrate whether HPC mediates brain ischemic tolerance via regulating the expression of piRNAs in CA1 after tGCI. We first reported that HPC alters the piRNAs expression profile, especially down‐regulates the tGCI‐induced expression of the top 5 piRNAs related to synaptic function in CA1. Additionally, we revealed that the down‐regulation of rno_piR_011022 mediated by HPC enhances synaptic plasticity in CA1 through inhibiting the NR2B‐PSD95 interaction, ultimately providing neuroprotection against tGCI.

## 2 Materials and Methods

### 2.1 Animals

All animal‐related experiments were conducted according to the *Animal Research: Reporting* In Vivo *Experiments* guidelines and were supervised by the Animals Care and Use Committee of Guangzhou Medical University. All efforts have been made to minimize suffering and the number of animals used. Detailed protocols are provided in Data S1.

### 2.2 Transient Global Cerebral Ischemia and Hypoxic Postconditioning

A four‐vessel occlusion model was established to induce tGCI [25]. HPC was conducted as follows: at 24 h after tGCI, rats were placed in a sealed plastic chamber of 9000 cm^3^ with a mixed gas composed of 8% O_2_ and 92% N_2_ for 2 h at a temperature of 23°C–25°C [26]. Detailed protocols are provided in Data S1.

### 2.3 Small RNA‐Sequence, Target Prediction and Functional Analyses

Small RNA‐sequence (RNA‐seq) service was offered by CloudSeq Biotech Ltd. Co. Detailed protocols are provided in Data S1.

### 2.4 Isolation of Total RNA and Reverse Transcription Quantitative Real‐Time Polymerase Chain Reaction

Using Trizol reagent, total RNA was extracted from the CA1 subregion. Reverse transcription real‐time quantitative PCR (RT‐qPCR) was performed according to the manufacturer's protocol. Detailed protocols are provided in Data S1.

### 2.5 Fluorescence In Situ Hybridization

Fluorescence in situ hybridization (FISH) was performed using Cy3‐labeled probes for rno_piR_negative control (NC), rno_piR_000618, rno_piR_009428, rno_piR_011022, rno_piR_014971, and rno_piR_017990 with a kit. Detailed protocols are provided in Data S1.

### 2.6 Western Blot and Co‐Immunoprecipitation

Western blot and immunoprecipitation were performed as previously described [27]. The primary antibodies, including PSD95, GRIN2B/NR2B, cleaved caspase 3, and glyceraldehyde 3‐phosphate dehydrogenase (GAPDH) were used. Detailed protocols are provided in Data S1.

### 2.7 Lentivirus Construction and Stereotaxic Injection

Plasmids containing the interference or overexpression sequence of rat rno_piR_011022 were designed by Genechem. Lentiviral administration was carried out as previously described [28]. Detailed protocols are provided in Data S1.

### 2.8 Assessment of Cellular Damage

Nissl and neuron‐specific nuclear protein (NeuN) staining were performed to evaluate CA1 neuronal damage [27]. Surviving cells were defined as well‐stained Nissl bodies, whereas damaged cells were either swollen with the loss of stainable Nissl material or necrotic with fragmenting deeply staining dendrites. The surviving cells in the CA1 pyramidal layer were quantitatively analyzed within three non‐repeated rectangular areas of 0.037 mm^2^. Detailed protocols are provided in Data S1.

### 2.9 Golgi‐Cox Staining and Imaging Analysis

Golgi‐Cox staining and imaging analysis were performed as previously described [8, 29]. Detailed protocols are provided in Data S1.

### 2.10 Neurobehavioral Assessments

Neurobehavioral assessments of rats were performed via the adhesive removal test [30], rotarod test [31], Morris water maze (MWM) [27] and novel object recognition (NOR) [32]. Detailed protocols are provided in Data S1.

### 2.11 Primary Hippocampal Neuron Culture, Lentivirus Transfection and Oxygen–Glucose Deprivation/Reoxygenation

Primary hippocampal neurons were cultured from 17‐ to 18‐day‐old rat embryos [33]. They were infected with sh‐*011022* or sh‐Con, or subjected to oxygen–glucose deprivation/reoxygenation (OGD/R). Detailed protocols are provided in Data S1.

### 2.12 Cell Counting Kit‐8 Assay

Cell counting kit‐8 was used to determine cell viability. Detailed protocols are provided in Data S1 [34].

### 2.13 Terminal Deoxynucleotidyl Transferase dUTP Nick End Labeling

The apoptosis was detected by terminal deoxynucleotidyl transferase dUTP nick end labeling (TUNEL) staining [35]. Detailed protocols are provided in the Data S1.

### 2.14 DiI Labeling of Neurons

Cultured neurons were labeled with 1, 1′‐dioctadecyl‐3, 3, 3′, 3′‐tetramethylindocarbocyanine perchlorate (DiI) in accordance with the instructions of the DiI staining Kit. Detailed protocols are provided in Data S1.

### 2.15 Luciferase Reporter Gene Assay

The luciferase reporter gene plasmid containing the putative binding site of NR2B was constructed and validated by Sangon Biotech Co. Ltd. Detailed protocols are provided in Data S1.

### 2.16 Immunofluorescence

To observe the colocalization of NR2B and PSD95, double‐fluorescent immunohistochemistry was performed as previously described [28]. Detailed protocols are provided in Data S1.

### 2.17 Statistical Analysis

Statistical analyses were performed using SPSS version 25.0. Data are expressed as mean ± SD. The normality of data and homogeneity of variance were checked. For normally distributed data, one‐way ANOVA and *t*‐tests were applied. The student two‐tailed *t*‐test was used for comparison between two groups. Multiple comparisons were conducted using one‐way ANOVA followed by Bonferroni's correction. Tamhane's T2 test was used for unequal variances. Nonparametric tests (Mann–Whitney *U* test for comparisons between two groups and Kruskal‐Wallis test among multiple groups) were used for abnormally distributed data and unequal variances. Values of *p* < 0.05 were considered statistically significant.

## 3 Results

### 3.1 HPC Alters the Expression Profiles of piRNAs in the CA1 Region After tGCI

To assess the expression profiles of piRNAs in tGCI with or without hypoxia, nine samples were sequenced using small RNA‐seq for each of the three groups (sham, tGCI 26 h, and HPC 0 h) with three biological replicates. The flow diagram in Figure S1 showed the time points for constructing tGCI and HPC models. Each sample's Q30 percentage, representing sequencing quality, exceeded 96% (Table S1). The RNA‐seq data sets of nine samples were subjected to principal component analysis (PCA), revealing that the samples of sham, tGCI 26 h, and HPC 0 h were clustered into separate groups (Figure S2).

**FIGURE 1:**
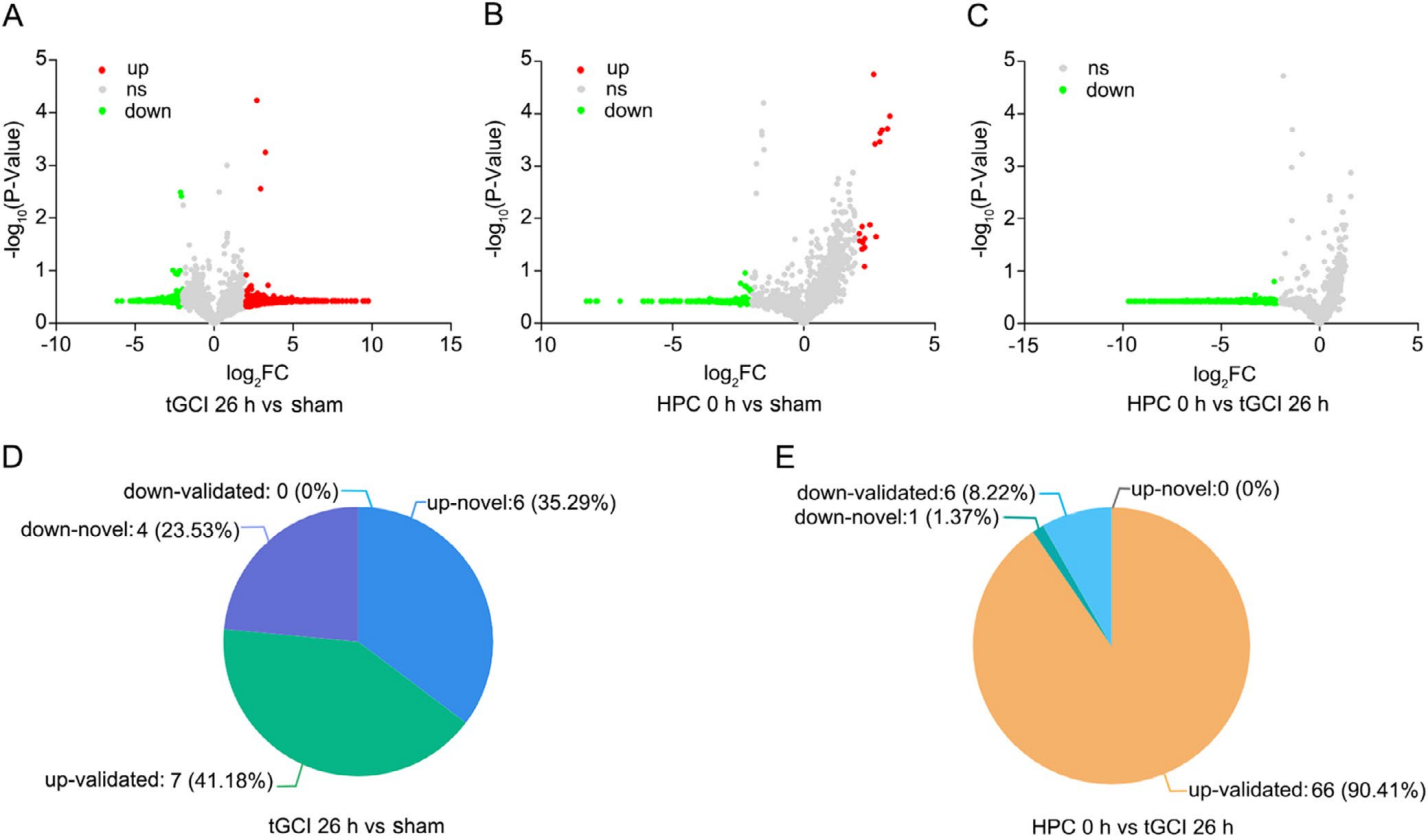
Differentially expressed piRNAs in CA1 after tGCI with or without hypoxia. Volcano plot shows the differential expressed piRNAs in CA1 for tGCI 26 h versus sham (A), HPC 0 h versus sham (B) and HPC 0 h versus tGCI 26 h (C) groups (*n* = 3, each group). Downregulation (green), upregulation (red) and no significance (gray). The number and percentage of differentially expressed piRNAs for tGCI 26 h versus sham (D) and HPC 0 h versus tGCI 26 h (E) are shown as a pie chart. h, hour; HPC, hypoxic postconditioning; sham, sham‐operated; tGCI, transient global cerebral ischemia; piRNAs, piwi‐interacting RNAs. Flow diagram for constructing tGCI and HPC models is shown in Figure S1. For assessing the quality of samples, please refer to Figure S2.

To demonstrate the overall distribution of differentially expressed piRNAs among sham, tGCI, and HPC groups, volcanic maps were constructed (Figure 1A–C). The number and percentage of piRNAs for tGCI 26 h versus sham (Figure 1D) and HPC 0 h versus tGCI 26 h (Figure 1E) are shown as pie charts. In comparison to sham, 17 piRNAs were differentially expressed in the tGCI group, including 13 up‐regulated and 4 down‐regulated piRNAs. Of those, 7 have been described in piRNAbank, and the other 10 were considered novel piRNA isoforms (Figure 1D and Table S2). However, in the HPC group, 66 were up‐regulated and 7 down‐regulated, including 1 novel piRNA molecule (Figure 1E and Table S3). Interestingly, through bioinformatic analysis, we found that the expressions of piRNAs (rno_piR_000618, rno_piR_009428, rno_piR_017990, rno_piR_014971, and rno_piR_011022) were significantly changed in both tGCI and HPC groups. These piRNAs were distributed across all chromosomes (chr) except for the sex chr. Y. Notably, the expressions of rno_piR_00618, rno_piR_011022, and rno_piR_017990 were up‐regulated more than 5‐fold in CA1 after tGCI, whereas HPC significantly reduced the expressions of these piRNAs (Table S4).

**FIGURE 2:**
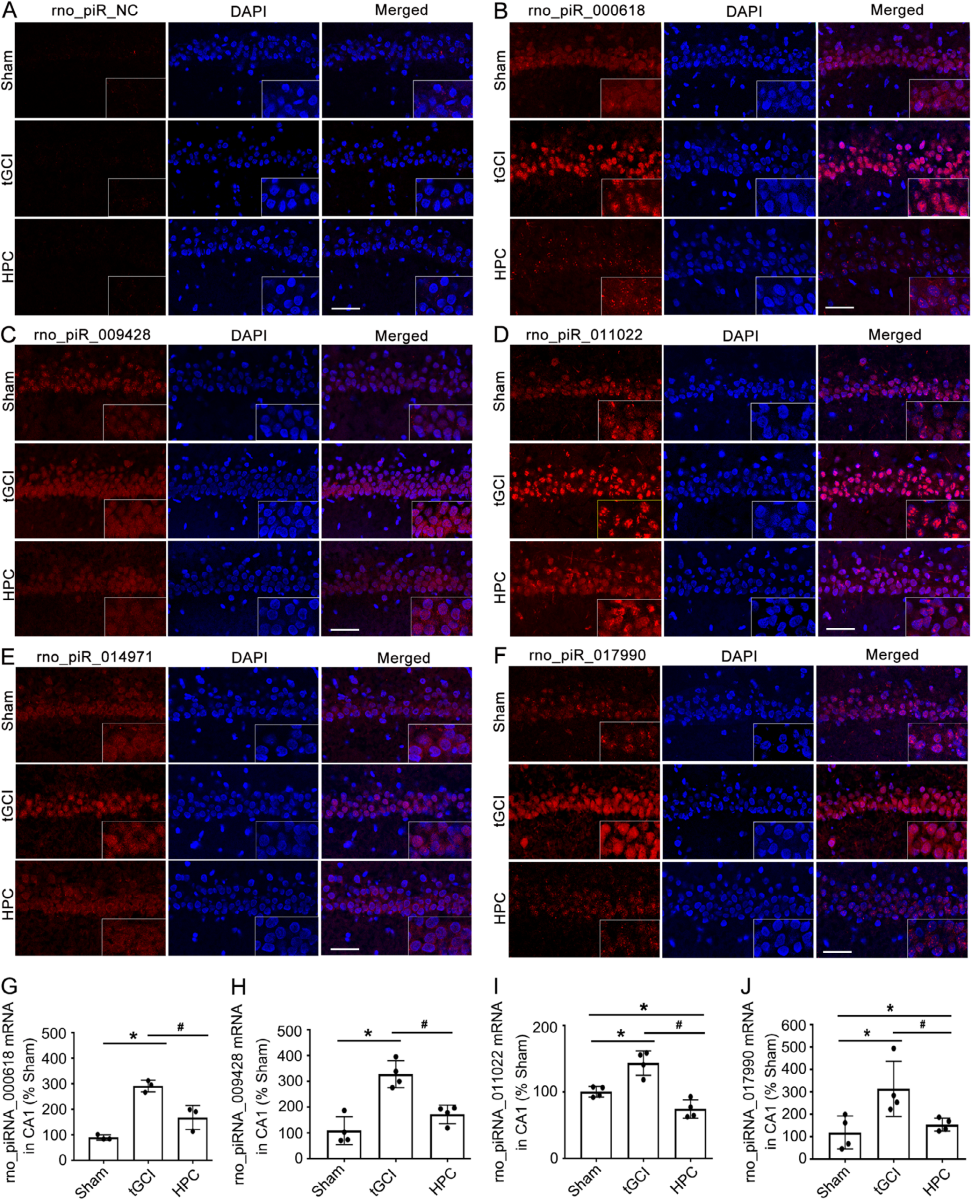
Validation of tGCI‐responsive piRNAs in CA1. (A) Negative control (NC) for rno_piRNA fluorescence in situ hybridization (FISH). (B–F) FISH of rno_piR_000618 (red), rno_piR_009428 (red), rno_piR_011022 (red), rno_piR_014971 (red), and rno_piR_017990 (red) and DAPI (blue) in CA1. (G–J) RT‐qPCR analysis of piRNAs‐rno_piR_000618, rno_piR_009428, rno_piR_011022, and rno_piR_017990 in CA1. Data are expressed as percentage of value of sham animals. Each bar represents the mean ± SD; *p < 0.05 vs. sham animals, ^#^ *p* < 0.05 vs. tGCI group. DAPI, 4′, 6‐diamidino‐2‐phenylindole; sham, sham‐operated; tGCI, transient global cerebral ischemia; HPC, hypoxic postconditioning; piRNAs, piwi‐interacting RNAs.

FISH and RT‐qPCR were used to validate the expression of the above 5 piRNAs selected from small RNA‐seq. The FISH, against rno_piR_000618, rno_piR_009428, rno_piR_011022, rno_piR_014971, and rno_piR_017990, demonstrated that the fluorescence signals of these piRNAs were mainly located in the cytoplasm in sham rats. However, at 26 h after tGCI, more fluorescence signals were co‐labeled with DAPI, suggesting nuclear translocation of piRNAs. With the treatment of HPC, the nuclear translocation of these piRNAs was partly blocked (Figure 2A–F). In agreement with the high expression profiles (Table S1), fluorescence signals of rno_piRNA_000618 and rno_piRNA_011022 were partly aggregated in the tGCI group (Figure 2B,D). Subsequently, RT‐qPCR was employed to validate the expression of aberrant piRNAs obtained by RNA‐seq (Table S4 and Figure 2G–J). As expected, HPC partially prevented the upregulation of rno_piR_000618, rno_piR_009428, rno_piR_011022, and rno_piR_017990 at the mRNA level after tGCI. Yet, only Ct values of rno_piRNA_014971 were near the assay detection limit (Ct > 40), indicating that its mRNA expression was under a low basal level and that it may not play a critical role after tGCI.

### 3.2 HPC Downregulates the Expression of Top 5 piRNAs Related to Synaptic Function in CA1 After tGCI

Next, we explored the target genes of the top 5 piRNAs, which were up‐regulated in the tGCI group while down‐regulated in the HPC group. As shown in Figure S3A, there were 8758, 8234, 8042, 6903, and 6284 target genes in rno_piR_011022, rno_piR_009428, rno_piR_000618, rno_piR_014971, and rno_piR_017990, respectively. Subsequently, STRING protein databases were used to generate a PPI network for the top 500 hub genes with the highest degrees of connectivity. Then, the co‐regulation networks of the top 10 hub genes of these piRNAs were constructed and visualized using Cytoscape software. As shown in Figure S3B–F, these piRNAs could regulate multiple target mRNAs related to neuroprotection against cerebral ischemia, such as epidermal growth factor receptor (EGFR), insulin‐like growth factor 1 (IGF1), cAMP response element binding protein 1 (CREB1), synaptotagmin (SYT) 1 and 2, and glutamate ionotropic receptor *N*‐Methyl‐D‐Aspartate type subunit (GRIN) 2A and 2B.

**FIGURE 3:**
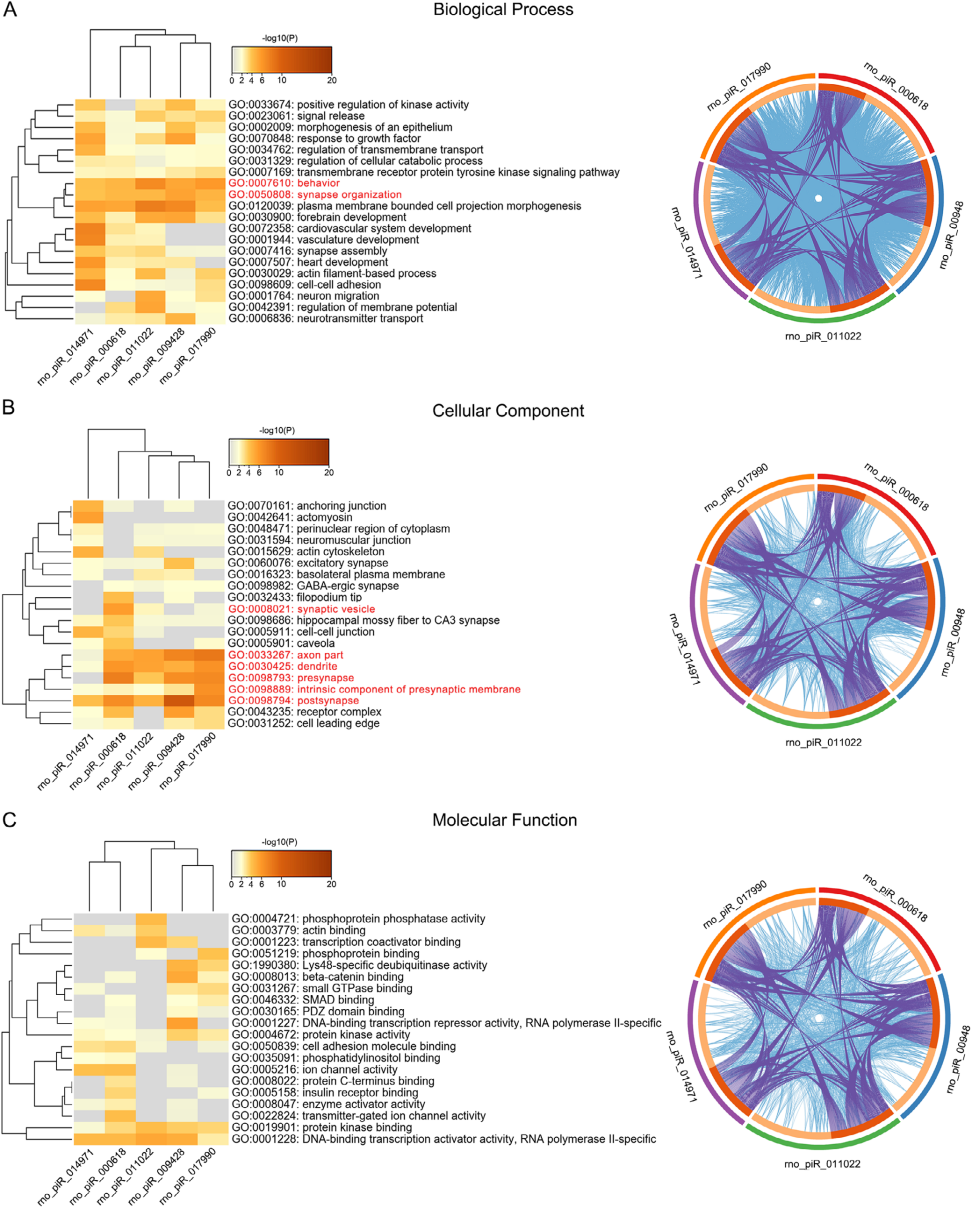
GO enrichment analyses originated from the target genes of 5 differentially expressed piRNAs. GO, gene ontology; piRNAs, piwi‐interacting RNAs.

In order to further elucidate the biological process, cellular component, and molecular function of the aforementioned piRNAs, gene ontology (GO) enrichment analyses were conducted. The top 20 statistically significant GO terms were shown in Figure 3. The GO annotation for biological processes suggested that rno_piR_014971, rno_piR_000618, rno_piR_011022, rno_piR_009428, and rno_piR_017990 were significantly enriched in genes associated with behavior and synapse organization (Figure 3A). As shown in Figure 3B, most of the significant GO terms were related to synaptic vesicle, axon part, dendrite, presynapse, intrinsic component of presynaptic membrane, and postsynapse in cellular component. Further, molecular function analysis of these genes showed that they were enriched in DNA‐binding transcription activator activity (RNA polymerase II‐specific, Figure 3C).

Since piRNAs can play a pivotal role in suppressing the expression of transposons in the brain [36], we predicted transposons related to 5 above‐mentioned piRNAs. As shown in Figure S4, there are mainly 4 distinct categories of transposable elements (TEs): short interspersed nuclear element (SINE), long interspersed nuclear element (LINE), simple sequence repeat (SSR), and long‐terminal repeat (LTR). To further characterize the functions of these piRNAs, we categorized the target transposons of each piRNA. The results showed that LTR, Satellite, SSR, Satellite, and LINE were predicted to be the most abundant target transposon types of rno_piR_000618, rno_piR_009428, rno_piR_011022, rno_piR_014971, and rno_piR_017990, respectively (Figure S4B–F). Of those, the predicted transposons of rno_piR_011022 were the most abundantly expressed and distributed across all chromosomes except chr. Y (Figure S5). As LINE is a type of transposon related to memory, rno_piR‐011022 was selected as the candidate piRNA for subsequent studies.

### 3.3 HPC Enhances Synaptic Plasticity in CA1 and Offers Neuroprotection Against tGCI via Down‐Regulation of rno_piR_011022

**FIGURE 4:**
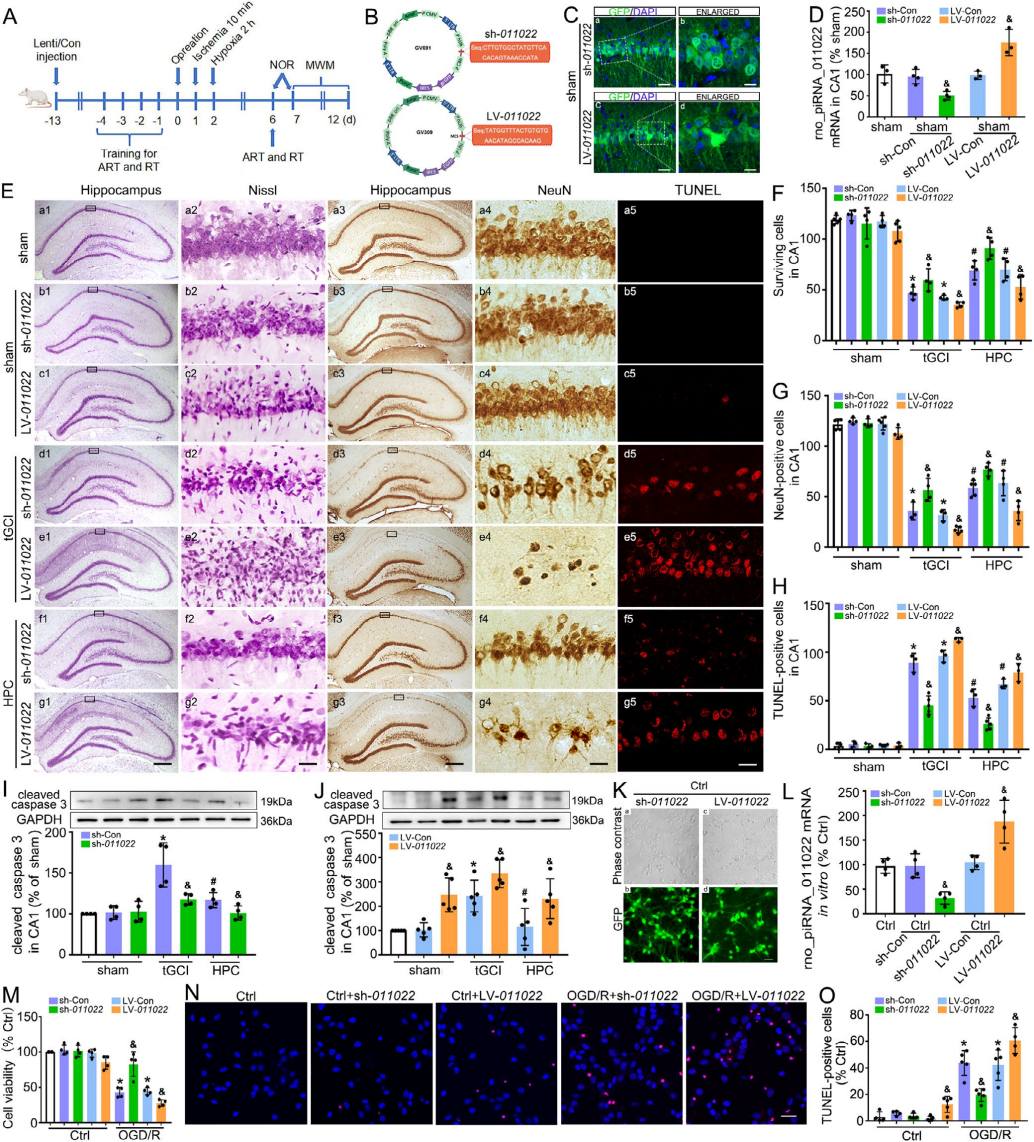
Downregulation of rno_piR_011022 protects neuron against tGCI in CA1. (A) Study design/timeline of bilateral injection with lentivirus vectors in the dorsal CA1 pyramidal layer. Neurobehavioral assessments of rats via adhesive removal test (ART), rotarod test (RT), novel object recognition (NOR) and Morris water maze (MWM). (B) Schema of the knockdown and overexpression lentiviral vector construct. (C) GFP expression was examined to determine the efficiencies of sh‐*011022* (a, b) and LV‐*011022* (c, d) transfection in CA1. Scale bar: A: 75 μm; b: 20 μm. (D) The knockdown and overexpression efficiencies of rno_piR‐011022 were determined with RT‐qPCR. ^&^ *p <* 0.05 vs. sham with corresponding control (sh‐Con or LV‐Con). (E) Representative microphotographs of cresyl violet staining, NeuN immunostaining and TUNEL staing in the hippocampus at 7 days after tGCI with sh‐*011022* or LV‐*011022* administration. sham (a1–a5); sham + sh‐*011022* group (b1–b5), injection with rno_piR_011022 shRNA lentivirus without ischemia or hypoxia; sham + LV‐*011022* group (c1–c5), injection with rno_piR_011022 overexpressed lentivirus without ischemia or hypoxia; tGCI + sh‐*011022* group (d1–d5), injection with rno_piR_011022 shRNA lentivirus before tGCI; tGCI+LV‐*011022* group (e1–e5), injection with rno_piR_011022 overexpressed lentivirus before tGCI; HPC + sh‐*011022* group (f1–f5), injection with rno_piR_011022 shRNA lentivirus before HPC; HPC + LV‐*011022* group (g1–g5), injection with rno_piR_011022 overexpressed lentivirus before HPC; Scale bar: A1–g1, a3–g3: 250 μm, a2–g2, a4–g4: 25 μm. Data are expressed as percentage of value of sham animals. Each bar represents the mean ± SD; (F–H) Quantitative analyses of surviving cells, NeuN‐positive cells and TUNEL‐positive cells in CA1. Data are expressed as the mean ± SD. *p < 0.05 vs. sham with corresponding control (sh‐Con or LV‐Con), ^#^ *p* < 0.05 vs. tGCI with same treatment, ^&^ *p* < 0.05 vs. the same group with corresponding control (sh‐Con or LV‐Con) (*n* ≥ 3 in each group). (I) Representative immunoblots of cleaved caspase 3 in CA1 at 26 h after reperfusion in tGCI or HPC group with or without sh‐*011022* administration. The histogram presents the quantitative analyses of cleaved caspase 3 in CA1. Data are expressed as the mean ± SD. *p < 0.05 vs. sham group with sh‐Con, ^#^ *p* < 0.05 vs. tGCI group with same treatment, ^&^ *p* < 0.05 vs. the same group with sh‐Con (*n* = 4 in each group). (J) Representative immunoblots of cleaved caspase 3 in CA1 at 26 h after reperfusion in tGCI or HPC group with or without LV‐*011022* administration. The histogram presents the quantitative analyses of cleaved caspase 3 in CA1. Data are expressed as the mean ± SD. *p < 0.05 vs. sham group with LV‐Con, ^#^ *p* < 0.05 vs. tGCI group with same treatment, ^&^ *p* < 0.05 vs. the same group with LV‐Con (*n* = 4 in each group). (K) Phase contrast (a, c) and fluorescent images (b, d, green) from primary hippocampal neurons transfected with sh‐*011022* (a‐b) or LV‐*011022* (c‐d). Scale bar: 25 μm. (L) Expression of rno_piR_011022 mRNA detected by qPCR after sh‐*011022* or LV‐*011022* transfection. Data are expressed as the mean ± SD. ^&^ *p* < 0.05 vs. Ctrl with corresponding control (sh‐Con or LV‐Con). (M) Cell viability was assessed with sh‐*011022* or LV‐*011022* transfection at 24 h after OGD/R injury. Data are expressed as the mean ± SD; *p < 0.05 vs. Ctrl with corresponding control (sh‐Con or LV‐Con), ^&^ *p* < 0.05 vs. OGD/R group with corresponding control (sh‐Con or LV‐Con) (*n* = 4 in each group). (N) Cell apoptosis was assessed with TUNEL staining (Red) with sh‐*011022* or LV‐*011022* transfection at 24 h after OGD/R injury. DAPI (blue) indicated nucleus. Scale bar: 25 μm. (O) Percentage of TUNEL positive cells was quantified. Data are expressed as the mean ± SD; *p < 0.05 vs. Ctrl with corresponding control (sh‐Con or LV‐Con), ^&^ *p* < 0.05 vs. OGD/R with corresponding control (sh‐Con or LV‐Con) (*n* ≥ 4 in each group). Ctrl, Control; d, day; DAPI, 4′, 6‐diamidino‐2‐phenylindole; GFP, Green fluorescent protein; HPC, hypoxic postconditioning; Lenti, lentivirus vector; LV‐*011022*, lentivirus‐overexpressed rno_piR_011022; LV‐Con, LV‐control; NeuN, neuronal nuclei; OGD/R, oxygen–glucose deprivation/reoxygenation; sh*‐011022*, lentivirus carrying shRNA against rno_piR_011022; sham, sham‐operated; sh‐Con, sh‐control; tGCI, transient global cerebral ischemia; TUNEL, TdT‐mediated dUTP Nick‐End Labeling. Results of the sh‐Con and LV‐Con groups are shown in Figure S6.

To verify the potential functions of rno_piR_011022 in the neuroprotection induced by HPC against tGCI, sh*‐011022* or LV‐*011022* were injected into bilateral CA1 stereotaxically to knock down or overexpress endogenous rno_piR‐011022 (Figure 4A). Schematic illustrations of the plasmid and the target site of rno_piR‐011022 were shown in Figure 4B. The efficacies of lentivirus transfection were verified using GFP (Figure 4C). The mRNA expressions of rno_piR‐011022 in CA1 of sham rats were effectively down‐regulated by sh‐*011022* and up‐regulated by LV‐*011022* (Figure 4D). As expected, the administration of sh‐*011022* or LV‐*011022* had no visible impact on the CA1 neurons of sham rats. However, sh‐*011022* administration markedly mitigated neuronal injury in CA1 after tGCI, manifested by a dramatic increase in the number of surviving and NeuN‐positive cells and a decrease in the number of TUNEL positive cells (Figure 4E–H and Figure S6A). Of note, an additive neuroprotective effect was observed with both HPC and sh‐*011022* treatments. Furthermore, sh‐*011022* administration abrogated tGCI‐induced increase of cleaved‐caspase 3 in CA1 (Figure 4I). Nevertheless, with LV‐*011022* administration, the neuronal injury was further aggravated compared with the tGCI group, accompanied by increased expression of cleaved‐caspase 3 in CA1 (Figure 4E–H,J). The neuroprotection induced by HPC was diminished. Further, primary hippocampal neurons were cultured and transfected using sh‐*011022* or LV‐*011022* (Figure 4K,L). In agreement with in vivo observations, sh‐*011022* administration elevated cell viability and alleviated apoptosis of hippocampal neurons under OGD/R conditions, while with LV‐*011022* treatment, opposite results were obtained (Figure 4M–O and Figure S6B).

**FIGURE 5:**
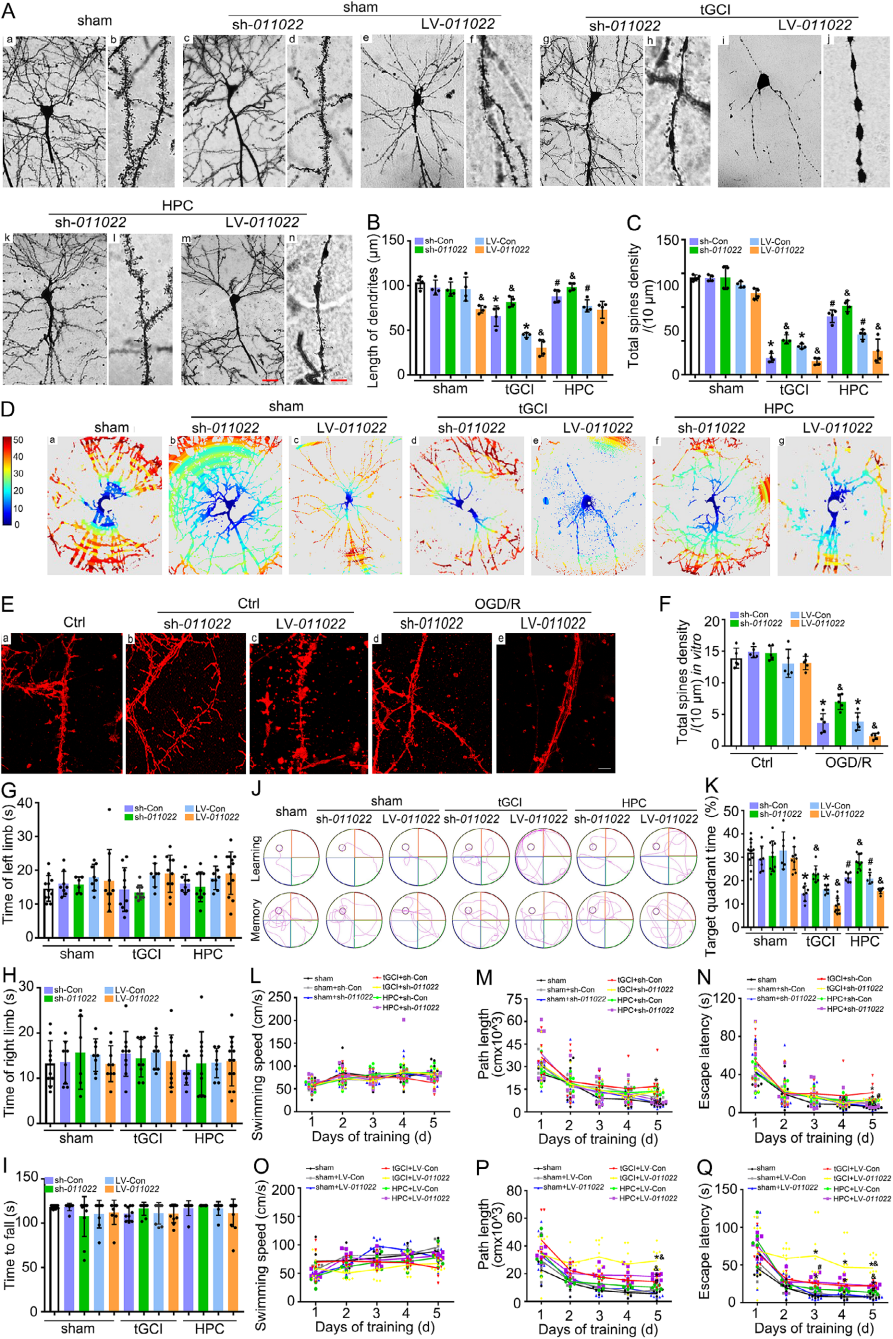
Downregulation of rno_piR_011022 enhances synaptic plasticity in CA1 and improves cognitive function after tGCI. (A) Representative reconstructions of CA1 pyramidal neurons using Golgi staining (a, c, e, g, i, k, m). Magnified views on the right show structural changes in dendritic spines during tGCI or HPC with sh‐*011022* or LV‐*011022* administration (b, d, f, h, j, l, n). sham group (a, b), sham + sh‐*011022* group (c, d); sham + LV‐*011022* group (e, f); tGCI+sh‐*011022* group (g, h), injection with rno_piR_011022 shRNA lentivirus before tGCI; tGCI+LV‐*011022* group (i, j), injection with rno_piR_011022 overexpressed lentivirus before HPC; HPC + sh‐*011022* group (k, l), injection with rno_piR_011022 shRNA lentivirus before HPC; HPC + LV‐*011022* group (m, n), injection with rno_piR_011022 overexpressed lentivirus before HPC. Scale bar: a, c, e, g, i, k, m, 60 μm; b, d, f, h, j, l, *n*, 5 μm. (B, C) Quantification of neuronal morphometric analyses, length of dendritic branch and density of dendrite spines. Data are expressed as the mean ± SD. *p < 0.05 vs. sham with corresponding control (sh‐Con or LV‐Con), ^#^ *p* < 0.05 vs. tGCI with same treatment, ^&^ *p* < 0.05 vs. the same group with corresponding control (sh‐Con or LV‐Con) (*n* = 4 in each group). (D) The graph represents the number of dendritic crossings along the concentric Sholl rings at different distances from the soma in each group. (E, F) Confocal images and quantification of dendrites in hippocampal neurons stained with DiI. Scale bars, 8 μm. Data are expressed as the mean ± SD. *p < 0.05 vs. Ctrl with corresponding control (sh‐Con or LV‐Con), ^&^ *p* < 0.05 vs. the same group with corresponding control (sh‐Con or LV‐Con) (*n* = 5 in each group). (G, H) The adhesive‐removal tests for the right and left limbs were performed, respectively. Data are expressed as the mean ± SD (*n* ≥ 6 in each group). (I) Average time to fall in the rotarod test after tGCI or HPC with sh‐*011022* or LV‐*011022* administration. Data are expressed as the mean ± SD (*n* ≥ 8 in each group). Morris water maze test was conducted. Representative path tracings in each quadrant during the training period (learning) and the probe trial (memory) (J). The percentage of time spent in the target quadrant to total time (30 s) was recorded at 12 days after tGCI (K). The swimming speed (L, O), The path length (M, P) and escape latency (N, Q) were recorded during the training period. Data are expressed as the mean ± SD. *p < 0.05 vs. sham with corresponding control (sh‐Con or LV‐Con), ^#^ *p* < 0.05 vs. tGCI with same treatment, ^&^ *p* < 0.05 vs. the same group with corresponding control (sh‐Con or LV‐Con) (*n* ≥ 5 in each group). sham, sham‐operated; tGCI, transient global cerebral ischemia; HPC, hypoxic postconditioning; sh‐Con, sh‐Control; sh*‐011022*, lentivirus carrying shRNA against rno_piR_011022; LV‐Con, LV‐Control; LV‐*011022*, lentivirus‐overexpressed rno_piR_011022; Ctrl, Control; OGD/R, oxygen–glucose deprivation/reoxygenation. Results of the sh‐Con and LV‐Con groups are shown in Figure S7.

To determine whether HPC enhances synaptic plasticity in CA1 after tGCI through downregulating rno_piR_011022, Golgi staining and DiI labeling were utilized, respectively. The results revealed that pyramidal neurons in CA1 after tGCI almost lost their dendrites, and the remainders were stunted, beaded dendrites devoid of spines, but they were partially restored when applied with HPC or sh‐*011022* treatments (Figure 5A and Figure S7A). Intriguingly, treatment with sh‐*011022* or HPC increased the number of dendritic branch intersections, dendrite length, and total spine density in CA1 neurons. Conversely, in the HPC groups with LV‐*011022* administration, these parameters representing the complexity of dendrites were significantly decreased, implying that LV‐*011022* eliminated the protection of HPC against tGCI (Figure 5A–D and Figure S7B). Meanwhile, with DiI labeling, we acquired high‐resolution images of changes in the dendritic spines with OGD/R treatment. Compared with the control, total spine density of primary hippocampal neurons with OGD/R was reduced, accompanied by a bead‐like appearance of neurons, which can be substantially reversed by sh‐*011022* treatment. However, LV‐*011022* further aggravated dendritic spine loss (Figure 5E,F and Figure S7C).

To confirm whether HPC improves neurological functions after tGCI through downregulating rno_piR_011022, neurobehaviors of rats were assessed. In the adhesive removal test, there was no significant difference in the time of removing sticky labels in rats of tGCI with or without hypoxia when compared with sham. Also, the treatment of sh‐*011022* or LV‐*011022* did not alter the time of removing the label in rats (Figure 5G,H). Similarly, in the rotarod test, sh‐*011022* or LV‐*011022* administration had no detectable impact on the fall time of rats both in tGCI and HPC groups (Figure 5I). In the MWM test, all groups of rats improved their ability to locate the platform after 5 days of training (Figure 5J and Figure S7D). However, the escape latency and path length in the tGCI group were longer than those of sham. It is worth noting that, compared to tGCI with sh‐Con or LV‐Con administration, HPC with sh‐Con or LV‐Con significantly shortened the path length on the 5th day of training and escape latency of rats from the 5th day of training (Figure 5J,M,N,P,Q). Moreover, in the probe phase, the rats in the tGCI group spent a shorter time in the target quadrant occupancy than that of sham and HPC ones (Figure 5K). To exclude the possibility that the spatial memory deficits were due to the difference in swimming ability, the swimming speed in the tGCI group was tested. As revealed in Figure 5L,O, no difference in the mean swimming velocity was observed among the experimental groups. Notably, the knockdown of rno_piR‐011022 with shRNA improved spatial learning and long‐term memory, with a shorter swimming path length and escape latency, and a longer time spent in the target quadrant occupancy in tGCI and HPC rats (Figure 5J,K,M,N). Conversely, the rats with LV‐*011022* injection in the HPC group spent a shorter time in the target quadrant occupancy than that of sham ones (Figure 5J,K,P,Q). However, in the NOR test, there were no significant differences in the duration of exploration for a novel object among sham, tGCI, and HPC groups (Figure S7E,F). Similarly, with sh‐*011022* or LV‐*011022* treatment, no difference was observed in the discrimination index of rats after tGCI with or without hypoxia (Figure S7E,F).

### 3.4 HPC Downregulates rno_piR_011022 Through Inhibiting NR2B‐PSD95 Interaction in CA1 After tGCI

**FIGURE 6:**
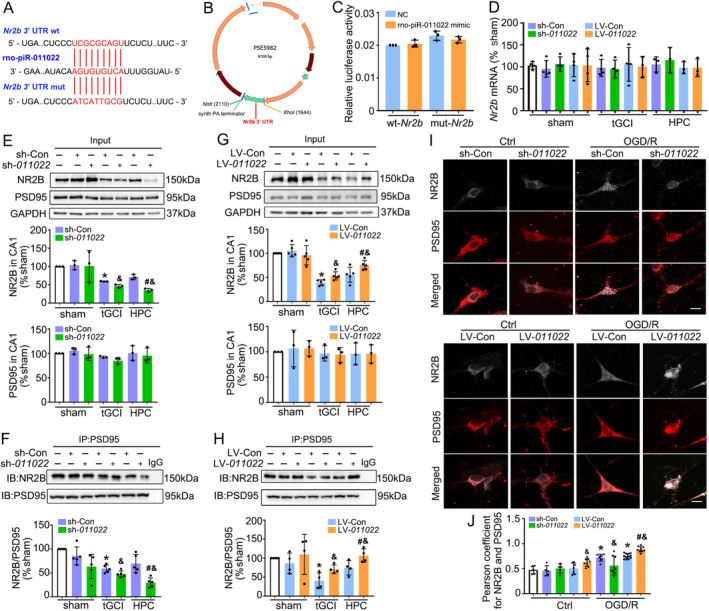
Downregulation of rno_piR_011022 protects neuron against tGCI by inhibiting NR2B‐PSD95 interaction in CA1. (A) Predicted sites of rno_piR_011022 binding to the 3′ UTR of wild type (wt)‐*Nr2b* and mut‐*Nr2b*, respectively. (B) The schematic of luciferase reporter vectors. (C) Luciferase activity was assessed by dual‐luciferase report assay (*n* = 3 in each group). (D) RT‐qPCR analysis of *Nr2b* mRNA in CA1 after tGCI with sh‐*011022* or LV‐*011022* administration. Data are expressed as percentage of value of sham animals. Each bar represents the mean ± SD. Western blot and immunoprecipitation assays showing the effects of sh‐*011022* (E, F) and LV‐*011022* (G, H) on the expression of NR2B and PSD95 and the interaction of NR2B‐PSD95 after tGCI with or without hypoxia. Data are expressed as the mean ± SD. *p < 0.05 vs. sham group with corresponding control (sh‐Con or LV‐Con), ^#^ *p* < 0.05 vs. tGCI group with same treatment, ^&^ *p* < 0.05 vs. the same group with corresponding control (sh‐Con or LV‐Con) (*n* ≥ 3 in each group). (I) The interaction between PSD95 (red) and NR2B (gray) in primary hippocampal neurons were observed under confocal microscope. Scale bars, 20 μm. (J) Co‐localization between PSD95 (red) and NR2B (gray) was determined by *Pearson* correlation coefficient and quantified. Data are expressed as the mean ± SD. *p < 0.05 vs. Ctrl group with corresponding control (sh‐Con or LV‐Con), ^#^ *p* < 0.05 vs. Ctrl group with same treatment, ^&^ *p* < 0.05 vs. the same group with corresponding control (sh‐Con or LV‐Con) (*n* ≥ 5 in each group). Ctrl, Control; HPC, hypoxic postconditioning; LV‐*011022*, lentivirus‐overexpressed rno_piR_011022; mut, Mutant; NR2B, *N*‐methyl‐D‐aspartate receptor 2B subunit; OGD/R, oxygen–glucose deprivation/reoxygenation; PSD95, post‐synaptic density protein 95; sh*‐011022*, lentivirus carrying shRNA against rno_piR_011022; sham, sham‐operated; sh‐Con, sh‐Control; tGCI, transient global cerebral ischemia; wt, Wild‐type.

To further test the potential function of rno_piR‐011022 in cerebral ischemia, the potential target genes of rno_piR‐011022 were predicted. Among those genes, NR2B was selected for further research because of its higher predictive scores and implications in neurotransmitter release at synapses. To explore the effect of rno_piR‐011022 on NR2B, using the bioinformatics software Targetscan, we found that the 3′‐UTR of *Nr2b* mRNA harbors one highly conserved potential binding site for rno_piR‐011022 (Figure 6A). Results from the dual luciferase reporter assay demonstrated that in cells carrying the mutant (mut)‐*Nr2b* plasmid, the luciferase activity did not differ significantly between wild type (wt)‐*Nr2b* and mut‐*Nr2b* groups (Figure 6B,C). No significant effects of sh‐*011022* or LV‐*011022* treatment on the mRNA level of *Nr2b* in CA1 were observed among these groups (Figure 6D). Whereas the expression of NR2B in CA1 after tGCI with or without hypoxia was down‐regulated by sh‐*011022* and up‐regulated by LV‐*011022* (Figure 6E,G). Further, we examined the NR2B‐PSD95 interaction after sh‐*011022* or LV‐*011022* treatment. Interestingly, the knockdown of rno_piR_011022 with shRNA induced a decreased NR2B‐PSD95 interaction in both tGCI and HPC groups (Figure 6F). In addition, a cumulative inhibitory effect of NR2B‐PSD95 interaction was observed when HPC and sh‐*011022* were combined without distinct alteration of PSD95 expression (Figure 6F). Nevertheless, the administration of LV‐*011022* exhibited contrary effects (Figure 6H). Consistently, double fluorescent immunohistochemistry demonstrated that with OGD/R treatment, there was a decrease in the co‐localization of NR2B and PSD95 in primary hippocampal neurons transfected with sh‐*011022*, whereas there was an increase with LV‐*011022* in comparison to their respective control groups (Figure 6I,J).

## 4 Discussion

In this study, the differentially expressed piRNAs in the hippocampal CA1 were identified after tGCI. We found that the expression profiles of piRNAs in CA1 were significantly changed after tGCI. HPC down‐regulated the expressions of the top 5 piRNAs associated with synaptic function after tGCI, including rno_piR_000618, rno_piR_009428, rno_piR_017990, rno_piR_014971, and rno_piR_011022. More importantly, HPC downregulated rno_piR_011022 in CA1 after tGCI through inhibiting the NR2B‐PSD95 interaction, thus enhancing synaptic plasticity, ameliorating neuronal injury, and improving the learning and memory functions of rats.

piRNAs are extensively expressed in a tissue‐specific way in somatic cells outside the germline [3]. To date, their functions have not been well characterized. There is evidence that piRNA expression profiles would be altered after ischemic stroke. Dharap et al. found that 105 piRNAs displayed modified expression in the cerebral cortex of rats following 1 h of MCAO when compared to the sham group. Of those, 25 were found to have 5‐fold changes (9 up‐ and 16 down‐regulation), suggesting the importance of piRNAs in cerebral ischemia [7]. Another study displayed significant differences in the abundance of piRNA expression among different brain regions, and the abundance of piRNA transcripts in the hippocampus was the highest [3]. It is well recognized that CA1 is the most sensitive to ischemia. Brief ischemia would result in neuronal apoptosis in the CA1 [37]. In this study, we found that tGCI significantly changed the expression profiles of piRNAs in CA1. Intriguingly, rno_piR_000618, rno_piR_009428, rno_piR_011022, rno_piR_014971, and rno_piR_017990 in the tGCI group were significantly upregulated. The changes in piRNA expression during cerebral ischemia might influence the activity of TEs and subsequent genomic changes. Similar to the cytoplasmic localization of piRNA (DQ541777) in cultured hippocampal neurons of mouse [38], we demonstrated a predominant cytoplasmic localization of these 5 piRNAs in hippocampal neurons of the sham groups by FISH. On the contrary, nuclear translocation of these piRNAs was observed after tGCI. Notably, HPC partially inhibited them from the cytoplasm into the nucleus. It is widely believed that the mature piRNA is transported to the nucleus to play a role in transposon silencing and gene expression [39]. Significantly, the present study revealed that HPC reduced the nuclear signal of these piRNAs and prevented their up‐regulation at the mRNA level from tGCI. As far as we know, this study is the first observation of the effect induced by tGCI on the nucleocytoplasmic distribution of piRNAs in CA1 neurons.

We previously reported that the loss of dendritic spines, the reduction in dendrite complexity, and the length of neurons in the dorsal hippocampal CA1 would impair the cognitive functions of rats after tGCI [8]. Considering that the differentially expressed piRNAs in CA1 are mainly clustered for axon guidance and synaptic function, which are thought to underlie higher cognitive functions, we further investigated the target genes of the above piRNAs, which were up‐regulated in the tGCI group while down‐regulated in the HPC group. We uncovered that these piRNAs could regulate multiple target mRNAs related to neuroprotection against cerebral ischemia. GO enrichment analyses further revealed that these piRNAs were significantly enriched in genes associated with behavior and synaptic structures and in DNA‐binding transcription activator activity. Of note, GRIN2B (*NR2B*) is crucial for synaptic plasticity and cognitive ability [40].

There were mainly four distinct categories of TEs. That is, SINE, LINE, SSR, and LTR in the prediction of transposons related to 5 piRNAs in this study. When further categorizing the target transposons of each piRNA, we found that LTR, Satellite, SSR, Satellite, and LINE were predicted to be the most abundant target transposon types of rno_piR_000618, rno_piR_009428, rno_piR_017990, rno_piR_014971, and rno_piR_011022, respectively. Specifically, the targets of rno_piRNA_011022 predominantly belonged to the LINE transposon family and were distributed across 21 chrs (1–20 and X). Initially, piRNAs were recognized as a critical mediators in maintaining normal homeostasis through repressing TE expression both in germline and somatic tissues such as brain [41, 42]. Transposons can be inserted into memory‐relevant loci [36]. Altered expression of piRNA might disrupt the normal transposon network, leading to pathophysiologic changes after stroke [7]. Bachiller et al. confirmed that LINE1 retrotransposition contributed to memory formation in the hippocampus [43]. These findings indicate that certain piRNAs, such as rno_piRNA_011022, may be involved in the cognitive impairment following cerebral ischemia.

Accumulating evidence suggests that the dysregulation of piRNA is associated with a wide range of biological processes, including cell proliferation, metastasis, and apoptosis [44, 45, 46]. However, most studies on cellular apoptosis regulated by piRNAs mainly focus on oncology [47, 48]. So far, the roles of piRNA in regulating neuronal apoptosis after cerebral ischemia have not been explored. Previously, we revealed that HPC induced neuroprotection by downregulating the activation of caspase‐3 and caspase‐9 to reduce apoptosis [49, 50]. Herein, we further demonstrated that HPC reduces the level of cleaved‐caspase 3 in CA1 following tGCI via down‐regulation of rno_piRNA_011022. Additionally, the suppression of rno_piRNA_011022 with sh‐*011022* alleviated neuronal injury after tGCI. In agreement with in vivo observations, sh‐*011022* elevated cell viability and decreased apoptotic neurons with OGD/R treatment. Whereas opposite results were obtained with LV‐*011022* treatment both in vivo and in vitro experiments. These data indicate that HPC offers neuroprotection against cerebral ischemia by suppressing rno_piRNA_011022 expression. To the best of our knowledge, this is the first study to reveal the involvement of rno_piRNA_011022 in neuronal damage after cerebral ischemia in rats.

Several studies have implicated that piRNA plays an essential role in regulating neurogenesis and synaptic plasticity by regulating gene expression at the genomic and transcriptional levels [12, 51, 52]. HPC could inhibit the expression of Piwil2 (a piRNA partner protein) in CA1 after tGCI, thereby reducing the ischemia‐induced damage to synaptic plasticity [8]. In this study, GO analysis of the top 5 piRNAs target genes set revealed moderate enrichment for synaptic genes. Several terms related to the structure and functions of synapses, such as axon part, dendrite, and synapse organization, were also observed. Of concern, the down‐regulation of rno_piRNA_011022 or HPC significantly alleviated ischemia‐induced synaptic injury. Under OGD/R conditions, the density of dendritic spine in hippocampal neurons was increased after sh‐*011022*. However, the mechanisms for the upregulation of dendritic spine abundance in hippocampal neurons after sh‐*011022* transfection remain unclear. Additionally, HPC and/or sh‐*011022* significantly improve cognition in rats after tGCI, while over‐expressed rno_piRNA_011022 with LV‐*011022* reversed the neuroprotective effects induced by HPC and sh‐*011022*.

In addition to silencing TEs, the piRNA pathway is also known to regulate protein‐coding genes in various developmental processes [53]. Of those, *GRIN2B* (*NR2B*), a hub gene in the hippocampus, is associated with synaptic plasticity and cognitive ability [14, 54]. Excessive activation of NMDARs, especially extrasynaptic GluN2B‐containing receptors, would cause neuronal death [55]. As a critical scaffolding protein during synaptogenesis and neurodevelopment, PSD95 can specifically bind to the C‐terminus of the NR2B through its PDZ domains [19]. The combination of both plays a crucial role in regulating downstream synaptic signaling and involves NMDAR‐mediated neuroexcitation and neurotoxicity [56, 57]. In this study, the knockdown of rno_piRNA_011022 resulted in a reduction of NR2B protein and a decreased NR2B‐PSD95 interaction in CA1 following tGCI. Whereas the overexpression of rno_piRNA_011022 presented the opposite results. Consistent with in vivo findings, the co‐localization of NR2B and PSD95 in primary hippocampal neurons was decreased with sh‐*011022*, while increased with LV‐*011022* following OGD/R. Of note, HPC enhanced the inhibitory effects of sh‐*011022* and provided neuroprotection against tGCI. However, research on the role of piRNAs in cerebral ischemia is just at the initial stage. The association between piRNAs and their epigenetic control is an ongoing endeavor in stroke research.

## 5 Summary

Our study presents the evidence that HPC induces neuroprotection against tGCI through regulating the expression of piRNAs in CA1. Specifically, for the first time, we demonstrated that rno_piRNA_011022, as a critical regulator, contributes to the synaptic plasticity and cognitive function of rats after tGCI. HPC downregulates tGCI‐induced expression of rno_piRNA_011022 in CA1, which inhibits the interaction of NR2B and PSD95, ultimately enhancing synaptic plasticity and offering neuroprotection against tGCI. Taken together, these observations provide the basis for targeting an inherent regulatory mechanism of rno_piRNA_011022 for therapeutic interventions to cerebral ischemia.

## Author Contributions

En Xu and Meiyan Chen conceived the study, designed the experiments, assembled all figures, and wrote the manuscript. Lixuan Zhan provided assistance for the design of this study. Meiyan Chen performed the experiments with the help of Shanshan Duan, Guorong Chai, Linhui Peng, and Weiwen Sun. Meiyan Chen and Shanshan Duan performed data analysis. All authors read and approved the final manuscript.

## Conflicts of Interest

The authors declare no conflicts of interest.

## Supporting information


**Figure S1.**Flow diagram for constructing tGCI and HPC models. HPC, hypoxic postconditioning; tGCI, transient global cerebral ischemia.
**Figure S2.** Principal Components Analysis (PCA). Principal component analysis was performed on nine samples according to the transcripts per million counts. Each dot on the plot represents one sample. Blue, green and red indicate sham, tGCI and HPC groups, respectively. HPC, hypoxic postconditioning; sham, sham‐operated; tGCI, transient global cerebral ischemia.
**Figure S3.** Target genes of 5 studied piRNAs. (A) Bar graph represents the number of target genes of top 5 differentially expressed piRNAs. (B–F) Visualization of top 5 differentially expressed piRNAs and their associated target genes with Cytoscape. piRNAs, piwi‐interacting RNAs.
**Figure S4.** The prediction of piRNA transposon. (A) Distribution of transposon classified statistics chart. (B–F) The transposon percentages of top five differentially expressed piRNAs are summarized in the pie chart. piRNAs, piwi‐interacting RNAs. For the characteristics of predicted transposons, see Figure S5.
**Figure S5.** Characteristics of predicted transposons. The tabulated and bar graph data show chromosomal location and predicted transposons number of top 5 piRNAs differentially expressed.
**Figure S6.** Downregulation of rno_piR_011022 protects neuron against tGCI in CA1. (A) Representative microphotographs of cresyl violet staining, NeuN immunostaining and TUNEL staining in the hippocampus at 7 days after tGCI with sh‐Con or LV‐Con administration. sham+sh‐Con group (a1–a5), injection with sh‐Con without ischemia or hypoxia; sham+LV‐Con group (b1–b5), injection with LV‐Con without ischemia or hypoxia; tGCI+sh‐Con group (c1–c5), injection with sh‐Con before tGCI; tGCI+LV‐Con group (d1–d5), injection with LV‐Con before tGCI; HPC + sh‐Con group (e1–e5), injection with sh‐Con before HPC; HPC + LV‐Con group (f1–f5), injection with LV‐Con before HPC; Scale bar: a1–f1, a3–f3: 250 μm, a2–f2, a4–f4: 25 μm. (B) Cell apoptosis was assessed with TUNEL staining (Red) with sh‐Con or LV‐Con transfection at 24 h after OGD injury. DAPI (blue) indicated nucleus. Scale bar: 25 μm. Ctrl, Control; DAPI:4′, 6‐diamidino‐2‐phenylindole; HPC, hypoxic postconditioning; LV‐*011022*, lentivirus‐overexpressed rno_piR_011022; LV‐Con, LV‐control; NeuN, neuronal nuclei; OGD/R, oxygen–glucose deprivation/reoxygenation; sh‐*011022*, lentivirus carrying shRNA against rno_piR_011022; sham, sham‐operated; sh‐Con, sh‐control; tGCI, transient global cerebral ischemia; TUNEL, TdT‐mediated dUTP Nick‐End Labeling.
**Figure S7.** Downregulation of rno_piR_011022 enhances synaptic plasticity in CA1 and improves cognitive function after tGCI. (A) Representative reconstructions of CA1 pyramidal neurons using Golgi staining (a, c, e, g, i, k). Magnified views on the right show structural changes in dendritic spines during tGCI or HPC with sh‐Con or LV‐Con administration (b, d, f, h, j, l). sham + sh‐Con group (a, b); sham + LV‐Con group (c, d); tGCI+sh‐Con group (e, f), injection with sh‐Con before tGCI; tGCI+LV‐Con group (g, h), injection with LV‐Con before HPC; HPC + sh‐Con group (i, j), injection with sh‐Con before HPC; HPC + LV‐Con group (k, l), injection with LV‐Con before HPC. Scale bar: a, c, e, g, i, k, 60 μm; b, d, f, h, j, l, 5 μm. (B) The graph represents the number of dendritic crossings along the concentric Sholl rings at different distances from the soma in sh‐Con and LV‐Con group. (C) Confocal images of dendrites in hippocampal neurons stained with DiI in sh‐Con and LV‐Con groups. Scale bars, 8 μm. (D) Representative path tracings in each quadrant during the training period (learning) and the probe trial (memory) in sh‐Con and LV‐Con groups. (E) Experimental setup of the novel object recognition (NOR) test and timeline of the experiment. (F) The NOR experiment was used to detect the ability of rats to distinguish new and old objects after tGCI or HPC with sh‐*011022* or LV‐*011022* administration. Data are expressed as the mean ± SD. (*n* ≥ 5 in each group). Ctrl, Control; HPC, hypoxic postconditioning; LV‐*011022*, lentivirus‐overexpressed rno_piR_011022; LV‐Con, LV‐Control; OGD/R, oxygen–glucose deprivation/reoxygenation; sh‐*011022*, lentivirus carrying shRNA against rno_piR_011022; sham, sham‐operated; sh‐Con, sh‐Control; tGCI, transient global cerebral ischemia.
**Table S1.** Q30 percentage of RNA‐seq in 9 samples.
**Table S2.** Characteristics of piRNAs differentially expressed in CA1 between tGCI and sham groups.
**Table S3.** Characteristics of piRNAs differentially expressed in CA1 between HPC and tGCI groups.
**Table S4.** Characteristics of the top 5 piRNAs differentially expressed in CA1 of rats after tGCI with or without hypoxia.

## Data Availability

The data that support the findings of this study are available from the corresponding author upon reasonable request.
