# Supplementary material for: Hypoxic Postconditioning Offers Neuroprotection Against Transient Cerebral Ischemia via Down‐Regulation of rno_piR_011022
Source: CNS Neurosci Ther. 2025 Feb 25;31(2):e70295. doi: 10.1111/cns.70295 (PMC11851155; doi:10.1111/cns.70295)
Supplement: Supplementary file 1 — Figure S1.Flow diagram for constructing tGCI and HPC models. HPC, hypoxic postconditioning; tGCI, transient global cerebral ischemia. Figure S2. Principal Components Analysis (PCA). Principal component analysis was performed on nine samples according to the transcripts per million counts. Each dot on the plot represents one sample. Blue, green and red indicate sham, tGCI and HPC groups, respectively. HPC, hypoxic postconditioning; sham, sham‐operated; tGCI, transient global cerebral ischemia. Figure S3. Target genes of 5 studied piRNAs. (A) Bar graph represents the number of target genes of top 5 differentially expressed piRNAs. (B–F) Visualization of top 5 differentially expressed piRNAs and their associated target genes with Cytoscape. piRNAs, piwi‐interacting RNAs. Figure S4. The prediction of piRNA transposon. (A) Distribution of transposon classified statistics chart. (B–F) The transposon percentages of top five differentially expressed piRNAs are summarized in the pie chart. piRNAs, piwi‐interacting RNAs. For the characteristics of predicted transposons, see Figure S5. Figure S5. Characteristics of predicted transposons. The tabulated and bar graph data show chromosomal location and predicted transposons number of top 5 piRNAs differentially expressed. Figure S6. Downregulation of rno_piR_011022 protects neuron against tGCI in CA1. (A) Representative microphotographs of cresyl violet staining, NeuN immunostaining and TUNEL staining in the hippocampus at 7 days after tGCI with sh‐Con or LV‐Con administration. sham+sh‐Con group (a1–a5), injection with sh‐Con without ischemia or hypoxia; sham+LV‐Con group (b1–b5), injection with LV‐Con without ischemia or hypoxia; tGCI+sh‐Con group (c1–c5), injection with sh‐Con before tGCI; tGCI+LV‐Con group (d1–d5), injection with LV‐Con before tGCI; HPC + sh‐Con group (e1–e5), injection with sh‐Con before HPC; HPC + LV‐Con group (f1–f5), injection with LV‐Con before HPC; Scale bar: a1–f1, a3–f3: 250 μm, a2–f2, a4–f4: 25 μm. (B [file CNS-31-e70295-s001.zip › cns70295-sup-0008-Supinfo.docx]

**Materials and methods**

**Animals**

Experiments were performed on adult male Wistar rats (7-8 week old) weighing between 250±30 g from the Animal Center of Southern Medical University (Guangzhou, China). All animal-related experiments were conducted according to the *Animal Research: Reporting In Vivo Experiments* guidelines and were supervised by the Animals Care and Use Committee of Guangzhou Medical University (Guangzhou, China). Rats were fed with adlibitum food and water and housed under standard temperature (21-23°C) in a 12-h light/dark, controlled environment. All efforts have been made to minimize suffering and the number of animals used.

In this study, 370 rats were used, 8 died during the surgery. Five rats that presented with convulsion and dyspnea after ischemia were excluded. In addition, 2 rats died after intrahippocampal injection of lentivirus during ischemia were also excluded.

**Transient global cerebral ischemia and hypoxic postconditioning**

As described previously^26^, a four-vessel occlusion model was established to induce tGCI. Briefly, rats were placed in the anesthesia induction chamber provided with 3-4% isoflurane mixed at 3 L/min. Delivered through a nose mask (SurgiVet, Waukesha, WI, United States), anesthesia was maintained with 2-3% isoflurane at 800 mL/min in air. Bilateral vertebral arteries were blocked by electrocauterization, and bilateral common carotid arteries (CCA) were isolated and assembled with a teflon/silastic occluding device without blocking blood flow. Subsequently, global ischemia was induced by occluding both CCA for 10 min at 24 h after surgery in awake rats. Afterwards, rats with mydriasis and loss of the righting reflex within 1 min were selected for the next experiments. Rectal temperature was maintained at 37-38°C throughout the experiment. The sham-operated (sham) rats received the same surgical procedures without 10-min occlusion of CCA.

At 24 h after tGCI, rats were treated with HPC^28^. Concisely, rats were placed in a sealed chamber of 9000 cm^3^ with continuous mixed-gas flow (8% O_2_ + 92% N_2_) at 23-25 °C for 2 h. Rats in sham, hypoxia-treated group were exposed to 2 h hypoxia at 24 h after sham operation without tGCI.

**Small RNA-sequence, target prediction and functional analyses**

Small RNA-sequence (RNA- seq) service was offered by CloudSeq Biotech Ltd. Co. (Shanghai, China). In brief, samples were collected from bilateral hippocampal CA1 region as genomic source (n=3 in each group). Total RNA of each sample was prepared with Trizol reagents (Invitrogen, Carlsbad, CA, USA), and the RNA quality and integrity were determined. 5’-and 3’-adaptors were ligated to the obtained small RNA. Reverse transcription followed by polymerase chain reaction (PCR) was used to create cDNA constructs. Then a fraction (about 150 bp) corresponding to approximate the adaptor-ligated constructs derived from the 30 nts piRNA fragment was excised and purified. With an Illumina HiSeq 2000 Sequencer, the purified and enriched libraries were sequenced. The raw data were refined by cutadapt software (v1.9.1). After filtering out low-quality and short reads (<15 nts), trimmed reads from all samples were pooled. The piano software was used to predict novel piRNAs. Using Novoalign software (v3.02.12) with at most one mismatch, trimmed reads were mapped to the merged rat piRNA databases (known piRNA from piRNABank plus the newly predicted piRNAs). piRNA-binding transposons and targets were predicted by miranda software (v3.3a). piRNA-targets networks were plotted by Cytoscape software (v2.8.0). The pathway and process enrichment analyses were carried out using Metascape (Metascape, <http://metascape.org).> The RNA-seq data have been deposited in GEO under accession no GSE163298.

**Isolation of total RNA and reverse transcription quantitative real-time polymerase chain reaction**

Using Trizol reagent (Invitrogen), total RNA was extracted from the CA1 subregion. Reverse transcription real-time quantitative PCR (RT-qPCR) was performed according to the manufacturer’s protocol. For each sample, 1 μg of RNA was reverse-transcribed into cDNA in a final volume of 20 μL with 1μL primer mix, 4 μL buffer and 1 μL enzyme mix (Takara, Shiga, Japan). The primers used are as follow: rno_piR_000618: 5’-CTGGCTGTCCTGGAACTCACT-3’(forward), 5’-AGTGCAGGGTCCGAGGTATT-3’(reverse), 5’-GTCGTATCCAGTGCAGGGTCCGAGGTATTCGCACTGGATACGACCTACAA-3’(RT Primer)，rno_piR_009428: 5’-ACAAAGTGAGTTCCAGGACAGC-3’(forward), 5’-AGTGCAGGGTCCGAGGTATT-3’(reverse),

5’-GTCGTATCCAGTGCAGGGTCCGAGGTATTCGCACTGGATACGACGCCCTG-3’(RT Primer), rno_piR_011022: 5’-CGTATGGTTTACTGTGTGAACATAGC-3’(forward), 5’-AGTGCAGGGTCCGAGGTATT-3’(reverse),

5’-GTCGTATCCAGTGCAGGGTCCGAGGTATTCGCACTGGATACGACCTTGTG-3’(RT Primer), rno_piR_014971: 5’-GCCAAGGCACTTCTGGCTTTCT-3’(forward), 5’-AGTGCAGGGTCCGAGGTATT-3’(reverse),

5’-GTCGTATCCAGTGCAGGGTCCGAGGTATTCGCACTGGATACGACGCCTTT-3’(RT Primer), rno_piR_017990: 5’-CAGCAAATACACCCAGGAGGA-3’(forward), 5’-AGTGCAGGGTCCGAGGTATT-3’(reverse), 5’-GTCGTATCCAGTGCAGGGTCCGAGGTATTCGCACTGGATACGACTTCTAC-3’(RT Primer)， U6:

5’-TGACACGCAAATTCGTGAAGCGTTC-3’(forward), 5’-CCAGTCTCAGGGTCCGAGGTATTC-3’(reverse) (Sangon Biotech, Shanghai, China). Afterwards, qPCR was performed in a final volume of 25 μL with 2 μL of RT product for cDNA amplification, and the annealing temperature was 60℃, which was conducted by LightCycler Fast-StartDNA Master SYBR Green 1 kit on a LightCycler 1.5 PCR machine (Roche Light Cycler 480, Germany). Data were analyzed using the comparative Ct method (2^-ΔΔCt^). Results were expressed as fold changes compared to Sham group.

**Fluorescence in situ hybridization**

Fluorescence in situ hybridization (FISH) was performed using Cy3-labeled probes (Gefan Biotechnology Co., Ltd , Shanghai, China) for rno_piR_negative control (NC) (5'-UUGUACUACACAAAAGUACUG-3'), rno_piR_000618 (5'-Cy3-CTACAAAGTGAGTTCCAGGACAGCCAGGG-3'), rno_piR_009428 (5'-Cy3-GCCCTGGCTGTCCTGGAACTCACTTTGTA-3'), rno_piR_011022 (5'-Cy3-CTTGTGGCTATGTTCACACAGTAAACCATA-3') ,rno_piR_014971 (5'-Cy3-GCCTTTCAGAAAGCCAGAAGTGCCTTG-3') and rno_piR_017990 (5'-Cy3-TTCTACTCCTCCTGGGTGTATTTGCTGCT-3'), with a kit (Gefan, Shanghai, China). Briefly, brain cryo sections (30 µm) were reactivated in hydrochloric acid at room temperature (RT) for 30 min, treated with proteinase K (37°C, 20 min) following blocked (37°C, 30 min), denatured (78°C, 2 min), dehydrated using graded ethanol (RT, 2 min) and then hybridized with probe working solution (probe: phosphate buffered saline (PBS)=1:50) in hybridization buffer overnight at 37°C. Hybridized sections were washed with 2×saline-sodium citrate (SSC) buffer (60°C, 1 min), 4×SSC (60°C, 30 min), and PBS (RT, 20 min). Finally, the coverslips were counterstained with 4’, 6-diamidino-2-phenylindole (DAPI, Solarbio, Beijing, China). Images were visualized under confocal microscope (SP8, Leica Microsystems, Wetzlar, Hessen, Germany).

**Western blot**

Rats were sacrificed at 26 h after reperfusion of tGCI with or without hypoxia (n=4 in each group). The brain samples were cut into 2 mm coronal slices with a brain matrix, and bilateral CA1 subregions were quickly dissected under a stereomicroscope. Proteins of CA1 were extracted and Western blot was performed as previously described^28^. The primary antibodies including PSD95 (1:5000; Abcam Cat# ab13552, RRID: AB_300453), GRIN2B/NR2B (1:2000; Proteintech, Cat# 21920-1-AP, RRID: AB_11232223), cleaved caspase 3 (1:1000; Proteintech, Cat# 19677-1-AP, RRID: AB_10733244), glyceraldehyde 3-phosphate dehydrogenase (GADPH; 1:10000; Proteintech, Cat# 60004-1-Ig, RRID: AB_2107436) were used. Quantification of the band density was carried out by densitometric analysis with image analysis software (Quantity One, Bio-Rad Laboratories, Hercules, CA). Relative optical densities of protein bands were calibrated with GADPH and normalized to those in Sham rats.

**Co-immunoprecipitation**

Co-immunoprecipitation (CoIP) assay was conducted as previously described^28^. An amount of 0.6 mg of protein was incubated with 12 mg of PSD95 antibody (diluted 1:50; Abcam, Cat# ab13552, RRID: AB_30045) overnight at 4°C. On the next day, the protein/antibody complex was added to packed protein G agarose beads (MilliporeSigma, Burlington, MA, USA, Cat# IP05). Following 4 h of incubation at 4°C, the complex was washed five times with cold PBS containing 1% Tween. Immunocomplexes were collected by centrifugation and eluted by boiling in loading buffer. The eluted protein samples (100 mg) were subjected to Western blot with GRIN2B/NR2B (1:2000; Proteintech, Cat# 21920-1-AP, RRID: AB_11232223) and PSD95 (1:5000; Abcam, Cat# ab13552, RRID: AB_300453).

**Lentivirus construction and stereotaxic injection**

Plasmids containing the interference or overexpression sequence of rat rno_piR_011022 (GenBank accession number DQ626472) and a NC sequence (CON137) were designed by Genechem (Shanghai, China). The sequence was inserted into AgeI and EcoRI sites of the hU6-MCS-Ubiquitin-EGFP-IRES-puromycin (GV280) lentiviral vector. Using Lipofectamine 2000 (Invitrogen), the shuttle vector and viral packaging system were cotransfected into HEK293T cells to produce recombinant lentiviruses. Then, HEK293T cells were used for viral infection. The infection efficiency was greater than 80%, as monitored with green fluorescent protein (GFP) expression. After 48 h of infection with shRNA against rno_piR-011022 (sh-*011022)* or rno_piR-011022 overexpression lentivirus (LV-*011022*), the cells were harvested, and total RNA was extracted to examine the expression of rno_piR_011022 mRNA. The titers were approximately 1×10^9^ TU/mL. Lentiviral administration was carried out as described previously^29^. A total of 5 μL volume (1.25 μL virus diluted by 3.75 μL enhanced solution) containing 1.25×10^9^ TU/mL of particles was injected into the bilateral CA1 region (3.5 mm posterior to bregma, 2.3 mm lateral tobregma, and 2.6 mm below the dura). The rats were arranged to recover for up to 14 days to enable sufficient gene expression.

**Assessment of cellular damage**

Nissl and neuron-specific nuclear protein (NeuN) staining were performed to evaluate CA1 neuronal damage^28^. Briefly, Nissl staining was performed with 0.1% cresyl violet (MilliporeSigma, Cat# C5042) for 7 min, and then the sections were dehydrated with 90% and 100% ethanoland and immersed into dimethylbenzene. Surviving cells were defined as well-stained Nissl bodies. Whereas damaged cells were either swollen with the loss of stainable Nissl material or necrotic with fragmenting deeply staining dendrites. NeuN staining was conducted via the singlelabel immunohistochemistry described above using anti-NeuN primary antibody (1:5000; Sigma-Aldrich, Cat# MAB377, RRID:AB_2298772). A light microscope was used to examine the sections after Nissl and NeuN staining (×660). The surviving cells or NeuN positive cells in the CA1 pyramidal layer were quantitatively analyzed within three non-repeated rectangular areas of 0.037mm^2^. Data were quantified bilaterally in sections from each brain and assessed blindly. Also, four sections for each animal were evaluated.

**Golgi-cox staining and imaging analysis**

Golgi-Cox staining and imaging analysis were performed following a previously published protocol^8^. Rat was first intracardially perfused with normal saline (NS) that was followed by a quick removal of the brain. The rapid Golgi staining was conducted with the FD Rapid Golgi Stain kit (FD Neurotechnologies Inc, Columbia, MD). Briefly, freshly dissected brains were immersed in solutions A and B for 2 w at RT and then transferred into solution C for 24 h at 4°C in the dark. The brains were cut into 100 μm using a vibratome (Leica VT1200S, Leica Microsystems, Wetzlar, Germany). Neurons were selected based on the following criteria. First, cells are located in the middle part of the section to avoid their branches being cut off. Second, cells are stained clearly and impregnated without interruption. And third, cells are spaced far apart from adjacent cells so that dendrites do not overlap.

Photomicrographs were generated with Leica Imaging System. Sequential 2D reconstructions of entire dendritic tree were generated using Image Pro 5.0. Total dentritic length of the pyramidal cells, including both basal and apical dendrites, was analyzed. Sholl analysis was used to quantify dendritic tracings^30^. Dendritic arborization was estimated by centering a transparent grid with concentric rings equidistant (10 μm) over the dendritic tree tracings and the number of ring intersections. To estimate the density of dendritic spines, high magnification (×100 immersion objective) tracings of a terminal segment were chosen at random from the basal and apical branches of the dendrite^30^. Basal and apical dendrites were selected based on the ratio 1:1. Spine density was estimated by counting the number of visible spines along the branch segment. Spine density was calculated as average number of spines per 10 μm of dendrite.

**Neurobehavioral assessments**

Neurobehavioral assessments of rats were performed via adhesive removal test^31^, rotarod test^32^, Morris water maze (MWM)^28^ and novel object recognition (NOR)^33^ by the investigators who were blinded to the experimental groups.

**Adhesive removal test**

The adhesive removal test was performed at 6 d after tGCI. Before surgical procedure, the animals were trained 1 trial/d for four consecutive days. In brief, rats were placed in a transparent box after a habituation period of 60 s; two adhesive tapes (200 mm^2^) were randomly placed with equal pressure onto each forepaw. The rat performance was assessed by measuring the time needed sense and to remove the adhesives. The time to remove each stimulus from forelimbs was recorded (2 trials/day)^31^.

**Rotarod test**

Rotarod test was examined at 6 d after tGCI. Before surgical procedure, all animals were trained for four consecutive days on a rotating cylinder till they stayed on it at least 2 min. The rotorod device comprises a metal frame equipped with a motorized, rotating assembly of rods and two transparent disks situated on opposite sides of the frame. Rats were allowed to keep stationary for a while at 0 rpm and the rotational speed was gradually increased to 25 rpm in 46 s-interval till the rats fell off from the cylinder. The trial would be ended if the rat gripped the device and spun around without attempting to walk on the rungs. Rats were tested for 2 trials/d and the mean duration on the device was recorded^32^.

**Morris water maze**

MWM was carried out as described previously with minor modifications^28^.The aquatic maze consists of a circular black pool (210 cm in diameter, 60 cm in height; Taimeng Technology Co., Ltd, Chengdu, China) filled with opaque water (20 cm in depth, 23±1℃), which was divided into four quadrants of equal size (Q1, Q2, Q3, and Q4). A transparent platform (escape platform, 10 cm in diameter) was placed 2.0 cm below the surface of the water in Q1.

Rats were placed in the pool facing toward the wall and every starting point was used in a different order each day. Starting positions were designated as Q1, Q2, Q3, or Q4. All rats were trained to find the hidden platform within a maximum time limit of 120 s and then allowed to remain on the platform for 15 s. If the rat failed to find the platform, it was guided to there, and the escape latency was recorded as 120 s. Throughout the training period, the platform remained in the same location. From the 7th day after tGCI, all rats were subjected to four consecutive trials per day at 10 min intervals for five consecutive days. For each trial, the path length, escape latency and swimming speed were recorded. Twenty-four hours after the end of a training period, a 30-sec probe trial without the platform was performed to assess the long-term memory. Each rat was placed in maze at a fixed starting point (the most distance to the platform position used during the training period). At 12 d after tGCI, the time in the target quadrant occupancy was also recorded.

To minimize variability in performance of rats, all tests were performed nearly at the same time each day in order. A video tracking system (SMART, Polyvalent video-tracking system, 35B73-C6C, PANLAB, Spain) was used to record the parameters of each test.

**Novel object recognition test**

Rats were allowed to adapt to a 1 m^3^ plastic container for 10 min at 24 h before the test. A 10 min training phase was utilized for rat to familiarize themselves with object A and B. The box was cleaned with 70% ethanol between each trail as well. Exactly 24 h after the training period, object B was replaced with a novel object C, and animals were granted 10 min to explore both objects. The behavior was recorded by a video camera positioned above the box. The recognition times (T) for objects A, B, and C were recorded and calculated. The discrimination index (TC/TC+TA) was analyzed^33^.

**Primary hippocampal neuron culture and lentivirus transfection**

Primary hippocampal neurons were prepared from 17- to 18-day-old rat embryos. Hippocampus were dissected and gently minced in Dulbecco's modification of Eagle's medium (DMEM) (Gibco, Grand Island, NY, USA, Cat# C11995500BT), then digested in 0.25% trypsin solution (Gibco, Cat# 25200056) at 37℃ for 15 min. Neurons were plated in culture dishes coated with 0.01% poly-D-lysine (Sigma Chemical Company, St Louis, Missouri, USA, Cat# P3513) and cultured for 7 d *in vitro* (DIV) in Neurobasal Medium (Gibco, Cat# 21103049) supplemented with 0.3 g/L L-Glutamine (Gibco, Cat# 25030149), 2% B27 (Gibco, Cat# 17504044) and 100 U/mL penicillin/streptomycin (Gibco, Cat# 15070063) 4 h after plating^34^.

The neurons were cultured for 8 d before experiments. For gene delivery into neurons, they were infected with lentiviruses captured expression vectors encoding rno_piR_011022 (sh-*011022*) or GFP (sh-Con) at a multiplicity of infection of 100 at DIV3.

**Oxygen-glucose deprivation/reoxygenation**

Primary cultured hippocampal neurons were subjected to oxygen-glucose deprivation/reoxygenation (OGD/R) *in vitro* to simulate ischemia-like conditions. Briefly, the culture medium was replaced by glucose-free DMEM (Gibco, Cat# 11966025), and then cells were placed into an oxygen-deprived (5% CO_2_ and 95% N_2_) incubator for 1.5 h at 37℃. The DMEM was then replaced with a regular medium and placed back into an incubator at normal conditions to undergo reoxygenation for 24 h.

**Cell counting kit-8 assay**

Cell counting kit-8 (CCK-8, Beyotime, China, Shanghai, Cat# C0039) was used to determine cell viability^35^. Totally 4×10^3^ cells were seeded in 96-well plates and allowed to adhere overnight. After incubation with lentiviruses for 72 h, 10 μL CCK-8 dye was added to each well, and cells were incubated for 1 h at 37℃. Subsequently, the absorbance was determined by a Gen5 microplate reader (BIOTEK, Vermont, USA) at 450 nm, and the relative cell viability was calculated as follows: relative cell viability=(mean experimental absorbance/mean control absorbance)×100%. The results were determined in triplicate experiments.

**Terminal deoxynucleotidyl transferase dUTP nick end labeling**

The apoptosis was detected blindly by terminal deoxynucleotidyl transferase dUTP nick end labeling (TUNEL) staining (Beyotime, Shanghai, China, Cat# C1089). Briefly, the frozen sections or primary hippocampal neurons were incubated in 0.5% Triton X‐100 (5 min) and TUNEL reaction mixture (60 min) at 37°C, followed by three washes in PBS. Cell nuclei were stained with DAPI. Images were acquired with a fluorescent microscope (SP8, Leica Microsystems) under high power magnification (×660). The number of TUNEL positive cells in CA1 was counted and quantified bilaterally in sections from each brain^36^. TUNEL-positive cells *in vitro* were analysed by counting the average number of cells (red) per 100 nuclei (blue) in three different visual fields.

**DiI Labeling of neurons**

Cultured neurons were labeled with 1, 1’-dioctadecyl-3, 3, 3’, 3’-tetramethylindocarbocyanine perchlorate (DiI) in accordance with the instruction of DiI staining Kit (Beyotime, Cat# C1991S). Briefly, neurons were left in 2.5 µM DiI for 30 min at 37.5°C to allow the dye to spread, and then washed three times with PBS and observed using confocal laser microscope (SP8, Leica Microsystems).

**Luciferase reporter gene assay**

The luciferase reporter gene plasmid containing the putative binding site of NR2B was constructed and validated by Sangon Biotech Co., Ltd. HEK293T cells were seeded into a 24-well plate at a density of 2×10^5^ cells/well and then cultured at 37 ℃ with 5% CO_2_ and 95% humidity. HEK293T cells were transfected with rno_piR-011022 mimics or NC. The luciferase activity was measured at 48 h after transfection with a Dual-Luciferase Reporter Assay System (Promega Corporation) using a fluorescence spectrophotometer (Infinite M200; Tecan Group, Ltd.), which was then normalized to Renilla luciferase activity.

**Immunofluorescence**

To observe the colocalization of NR2B and PSD95, double-fluorescent immunohistochemistry was performed as described previously^29^. Antibodies used in these studies included NR2B (1:100; Proteintech, Cat# 21920-1-AP, RRID: AB_11232223), PSD95 (1:500; Abcam, Cat# ab13552, RRID: AB_300453), Cy3-conjugated goat anti-Rabbit IgG antibody (1:100; Millipore, Cat# AP132C, RRID:AB_92489), and FITC-conjugated goat anti-Mouse IgG antibody (1:100; Abcam, Cat# ab150113, RRID:AB_2576208). Fluorescent images were captured under confocal laser microscope (SP8, Leica Microsystems). The *Pearson* colocalization coefficient for the NR2B and PSD95 stacked signal was calculated.

**Statistical analysis**

Statistical Package for Social Sciences Software for Windows (SPSS, Inc., Chicago, IL, version 25.0) was used for statistical analyses. Data were expressed as mean±SD. All data were checked by normal distribution and homogeneity of variance, respectively. When the data were normally distributed, one-way ANOVA and *t*-test were applied. The student two-tailed *t*-test was used for comparison between two groups. Multiple comparisons were conducted using one-way ANOVA followed by Bonferroni's correction. Tamhane's T2 test was used for unequal variances. Nonparametric tests (Mann-Whitney U test for comparisons between two groups and Kruskal-Wallis test among multiple groups) were used for abnormal distributed data and unequal variances. Values of *p*< 0.05 were considered statistically significant.
