# Supplementary material for: Hypoxic Postconditioning Offers Neuroprotection Against Transient Cerebral Ischemia via Down‐Regulation of rno_piR_011022
Source: CNS Neurosci Ther. 2025 Feb 25;31(2):e70295. doi: 10.1111/cns.70295 (PMC11851155; doi:10.1111/cns.70295)
Supplement: Supplementary file 1 — Figure S1.Flow diagram for constructing tGCI and HPC models. HPC, hypoxic postconditioning; tGCI, transient global cerebral ischemia. Figure S2. Principal Components Analysis (PCA). Principal component analysis was performed on nine samples according to the transcripts per million counts. Each dot on the plot represents one sample. Blue, green and red indicate sham, tGCI and HPC groups, respectively. HPC, hypoxic postconditioning; sham, sham‐operated; tGCI, transient global cerebral ischemia. Figure S3. Target genes of 5 studied piRNAs. (A) Bar graph represents the number of target genes of top 5 differentially expressed piRNAs. (B–F) Visualization of top 5 differentially expressed piRNAs and their associated target genes with Cytoscape. piRNAs, piwi‐interacting RNAs. Figure S4. The prediction of piRNA transposon. (A) Distribution of transposon classified statistics chart. (B–F) The transposon percentages of top five differentially expressed piRNAs are summarized in the pie chart. piRNAs, piwi‐interacting RNAs. For the characteristics of predicted transposons, see Figure S5. Figure S5. Characteristics of predicted transposons. The tabulated and bar graph data show chromosomal location and predicted transposons number of top 5 piRNAs differentially expressed. Figure S6. Downregulation of rno_piR_011022 protects neuron against tGCI in CA1. (A) Representative microphotographs of cresyl violet staining, NeuN immunostaining and TUNEL staining in the hippocampus at 7 days after tGCI with sh‐Con or LV‐Con administration. sham+sh‐Con group (a1–a5), injection with sh‐Con without ischemia or hypoxia; sham+LV‐Con group (b1–b5), injection with LV‐Con without ischemia or hypoxia; tGCI+sh‐Con group (c1–c5), injection with sh‐Con before tGCI; tGCI+LV‐Con group (d1–d5), injection with LV‐Con before tGCI; HPC + sh‐Con group (e1–e5), injection with sh‐Con before HPC; HPC + LV‐Con group (f1–f5), injection with LV‐Con before HPC; Scale bar: a1–f1, a3–f3: 250 μm, a2–f2, a4–f4: 25 μm. (B [file CNS-31-e70295-s001.zip › cns70295-sup-0009-Tables.pdf]

Sample	Q30
sham-1	97.95%
sham-2	98.05%
sham-3	97.19%
tGCI 26 h-1	97.74%
tGCI 26 h-2	97.91%
tGCI 26 h-3	96.72%
HPC 0 h-1	97.79%
HPC 0 h-2	97.97%
HPC 0 h-3	98.49%

176x228mm (300 x 300 DPI)

piRNA name	piRNA-sequence	Alteration	Fold Change	*p*-value
novel_10364_x329	UAAUGGUUCGUUUGUAAAAAAAAAAAAA	down	-4.225	0.004
novel_11128_x300	UAAUGGUUCGUUUGUAAAAAAAAAAAA	down	-4.370	0.003
novel_11554_x284	UAAUGGUUCGUUUGUAAAAAAAAAAAAAA	down	-3.903	0.006
novel_196_x97317	AGAAUUGUGGCUGGACAUC	down	-2.974	0.032
rno_piR_000553	CCAAGACUACAUGCCAUCAUCGGAGCCCAAUU	up	1.571	0.047
rno_piR_000618	CCCUGGCUGUCCUGGAACUCACUUUGUAG	up	7.667	0.003
rno_piR_009428	UACAAAGUGAGUUCCAGGACAGCCAGGGC	up	1.240	0.003
rno_piR_011022	UAUGGUUUACUGUGUGAACAUAGCCACAAG	up	9.439	0.001
rno_piR_014971	CAAGGCACUUCUGGCUUUCUGAAAGGC	up	1.753	0.001
rno_piR_017990	AGCAGCAAAUACACCCAGGAGGAGUAGAA	up	6.516	0.000
rno_piR_025697	UCCACUACAGAUUGAGUGAGGUCUCCCUGA	up	3.333	0.041
novel_11018_x303	UGGUAGAAUUCUCGCCUGCCACGCGGGAGGCC	up	1.783	0.019
novel_11549_x284	UGGUAGAAUUCUCGCCUGCCACGCGG	up	1.758	0.029
novel_12299_x261	AGCUACAUCUGGCUACUGGGUCUCA	up	1.552	0.034
novel_12954_x243	UGGUAGAAUUCUCGCCUGCCACGCGGGAGGC	up	1.783	0.019
novel_4693_x1000	UGGUAGAAUUCUCGCCUGCCACGCGGGAG	up	1.754	0.023
novel_9407_x375	UGGUAGAAUUCUCGCCUGCCACGCGGGAGG	up	1.783	0.019

209x297mm (300 x 300 DPI)

piRNA name	piRNA-sequence	Alteration	Fold Change	*p*-value
rno_piR_000618	CCCUGGCUGUCCUGGAACUCACUUUGUAG	down	-2.674	0.011
rno_piR_009428	UACAAAGUGAGUUCCAGGACAGCCAGGGC	down	-3.598	0.000
rno_piR_011022	UAUGGUUUACUGUGUGAACAUAGCCACAAG	down	-1.490	0.023
rno_piR_014971	CAAGGCACUUCUGGCUUUCUGAAAGGC	down	-1.862	0.001
rno_piR_017990	AGCAGCAAAUACACCCAGGAGGAGUAGAA	down	-2.623	0.000
rno_piR_022116	UGAAAUAAAACAAAACUGUAAGAAGCCCAG	down	-2.700	0.001
novel_14036_x218	AUUUCGGUUGGGAAAAAAAAAAAAAAAAAAAA	down	-3.393	0.046
rno_piR_002430	UGAACUCAGGACCUCUGGAAGAGCAGA	up	2.000	0.000
rno_piR_007332	UGUGAUUUGAACUCAGGACCUCUGGAA	up	2.000	0.047
novel_10100_x342	UUCAAGAGGGCGUGAAACCGU	up	1.800	0.016
novel_10159_x339	GCGUGGAGCCGGGCGUGGAA	up	3.000	0.001
novel_102_x257826	UCAGUGCAUCACAGAACUU	up	2.000	0.000
novel_10834_x311	CCACUGCCCCAGGUGCUGCU	up	2.167	0.025
novel_10983_x305	AUCGGAUCCGUCUGAGCUUGG	up	1.538	0.037
novel_11380_x290	UGGCUCGGGGGGGGCGGCGUCA	up	2.000	0.024
novel_11397_x289	UGGUGUAAUGGUUAGCACUCUG	up	2.000	0.000
novel_11655_x281	UCGGUCGAUCGGUCGGUCGGU	up	1.394	0.034
novel_11687_x280	CGUGGAGCCGGGCGUGGAAUG	up	2.250	0.007
novel_11777_x277	AGAGGCUUAUAGCUCUAA	up	1.800	0.016
novel_11876_x274	UAACCGAAAGGUUGGUGGUUCGAGCCCACCCAGGGA	up	2.000	0.000
novel_11915_x273	UAGUGGUUAGUACUCUGC	up	1.800	0.016
novel_12712_x249	UCGGAUCCGUCUGAGCUG	up	2.000	0.000
novel_12841_x246	CGGUGCUGGUGGAGCAGUGAGC	up	2.111	0.034
novel_13611_x227	GUGUAAUGGUUAGCACUCUGGA	up	2.200	0.013
novel_13618_x227	AUUGAUCAUCGACACUUC	up	2.333	0.016
novel_13678_x225	GUUCGGGGUUCUCCUCCGCGUCGG	up	2.200	0.013
novel_137_x176898	CAUCAUCGUCUCAAAUGA	up	1.800	0.016
novel_13877_x222	CGUGGAGCCGGGCGUGGAA	up	2.250	0.007
novel_138_x176465	AGCUACAUUGUCUGCUGGG	up	1.800	0.016
novel_13950_x220	UUCCGGCGGCGUCCGGUGAGCUCUC	up	3.000	0.000
novel_14654_x205	GUAACCCGUUGAACCCCAUUCGU	up	2.000	0.047
novel_2021_x3387	UAUAGUGGUUAGUACUCUGC	up	1.800	0.016
novel_2194_x2976	GAAUGUUGCUCGGUGAACC	up	2.000	0.000
novel_2365_x2647	UCGGAUCCGUCUGAGCGU	up	2.000	0.000
novel_2844_x2045	UGUAAUGGUUAGCACUCUGGAC	up	2.000	0.024
novel_2932_x1953	UAUUCAAACGAGAACUUUGAAGGCCGA	up	2.000	0.047

209x297mm (300 x 300 DPI)

piRNA name	piRNA-sequence	Alteration	Fold Change	*p*-value
novel_2954_x1929	GAGAAUUGUGGCUGGACA	up	2.000	0.000
novel_311_x55490	AUCGGAUCCGUCUGAGCU	up	2.000	0.000
novel_3664_x1409	UUGGAUUGUUCACCCACUAA	up	2.250	0.007
novel_367_x42763	UCUACAGUGCACGUGUCU	up	2.000	0.000
novel_3813_x1335	CUGGACUGAGGCUCCUUGA	up	1.800	0.016
novel_4128_x1183	ACAGUAGUCUGCACAUUGG	up	2.000	0.000
novel_433_x32354	UCACAGUGAACCGGUCUCUUU	up	1.163	0.018
novel_4409_x1080	UGACCGAUUUCUCCUGGUGU	up	2.000	0.000
novel_4459_x1064	UUCAAGAGGGCGUGAAACCGUUAA	up	2.000	0.035
novel_44_x739592	UCGGAUCCGUCUGAGCUUGG	up	1.428	0.004
novel_5018_x906	CAUCGGAUCCGUCUGAGC	up	2.000	0.000
novel_5034_x902	CUCGGAUCCGUCUGAGCU	up	2.000	0.000
novel_5399_x818	GUGUAAUGGUUAGCACUCUGGAC	up	2.200	0.013
novel_54_x580814	AGAAUUGUGGCUGGACAU	up	2.000	0.047
novel_5564_x783	CAUCGGAUCCGUCUGAGCU	up	2.000	0.000
novel_5778_x744	UCGGAUCCGUCUGAGCUC	up	2.000	0.000
novel_5995_x703	UUCGGGGUUCUCCUCCGCGUCGG	up	1.800	0.016
novel_6337_x655	AAUAUUCAAACGAGAACUUUGAAGGCCGA	up	2.000	0.047
novel_654_x18599	UAUUGCACUCGUCCCGGCC	up	1.800	0.016
novel_6687_x607	UGGCGUGGAGCCGGGCGUGGAAUG	up	3.000	0.004
novel_6847_x586	CAGCAGCAAUUCAUGUUUU	up	1.800	0.016
novel_7189_x542	AGGACACAUUGAUCAUCGACAC	up	2.000	0.026
novel_7326_x530	UGGCGUGGAGCCGGGCGUGGAA	up	2.500	0.013
novel_7485_x515	UCGGAUCCGUCUGAGCCU	up	2.000	0.000
novel_7586_x506	CUCGGAUCCGUCUGAGCUUGG	up	1.431	0.004
novel_7865_x481	AUAUUCAAACGAGAACUUUGAAGGCCGA	up	2.000	0.047
novel_8187_x453	UCGUCGCGCCGUCGGGCC	up	2.500	0.022
novel_8246_x449	UAUUCAAACGAGAACUUUGAAG	up	2.000	0.047
novel_8470_x433	UUGUAAACCAGGGGUCGCG	up	2.000	0.047
novel_8560_x427	GGCGUGGAGCCGGGCGUGGAAUG	up	3.000	0.001
novel_8901_x405	GGCGUGGAGCCGGGCGUGGAA	up	3.000	0.001
novel_8954_x402	GCGUGGAGCCGGGCGUGGAAUG	up	3.000	0.001
novel_9043_x397	UGUAAUGGUUAGCACUCUGGA	up	2.000	0.024
novel_9073_x395	UUGGAUUGUUCACCCACUA	up	2.250	0.007
novel_9412_x375	GUGUGUCAGGAUGGCCGAGUGG	up	2.000	0.000
novel_9663_x362	AACGCGCCCGAUCUCGUCUGAU	up	2.429	0.039
novel_9960_x348	CUGGUUUCACAUGGUGGCUUA	up	2.000	0.047

209x297mm (300 x 300 DPI)

piRNA name	piRNA sequence	Chromsomal location	tGCI 26 h vs sham up-fold change	HPC 0 h vs tGCI 26 h down-fold change	NCBI gene ID
rno_piR_000618	CCCUGGCUGUCCUGGAACUCACUUUGUAG	1-20, X	7.67	-2.67	DQ603196
rno_piR_009428	UACAAAGUGAGUUCCAGGACAGCCAGGGC	1-20, X	1.24	-3.60	DQ622691
rno_piR_011022	UAUGGUUUACUGUGUGAACAUAGCCACAAG	2-8, 10,11,13,18,19,X	9.44	-1.49	DQ626472
rno_piR_014971	CAAGGCACUUCUGGCUUUCUGAAAGGC	8	1.75	-1.86	DQ732100
rno_piR_017990	AGCAGCAAAUACACCCAGGAGGAGUAGAA	7	6.52	-2.62	DQ736825

209x297mm (300 x 300 DPI)
