# Supplementary figures and images for: Hypoxic Postconditioning Offers Neuroprotection Against Transient Cerebral Ischemia via Down‐Regulation of rno_piR_011022
Source: CNS Neurosci Ther. 2025 Feb 25;31(2):e70295. doi: 10.1111/cns.70295 (PMC11851155; doi:10.1111/cns.70295)

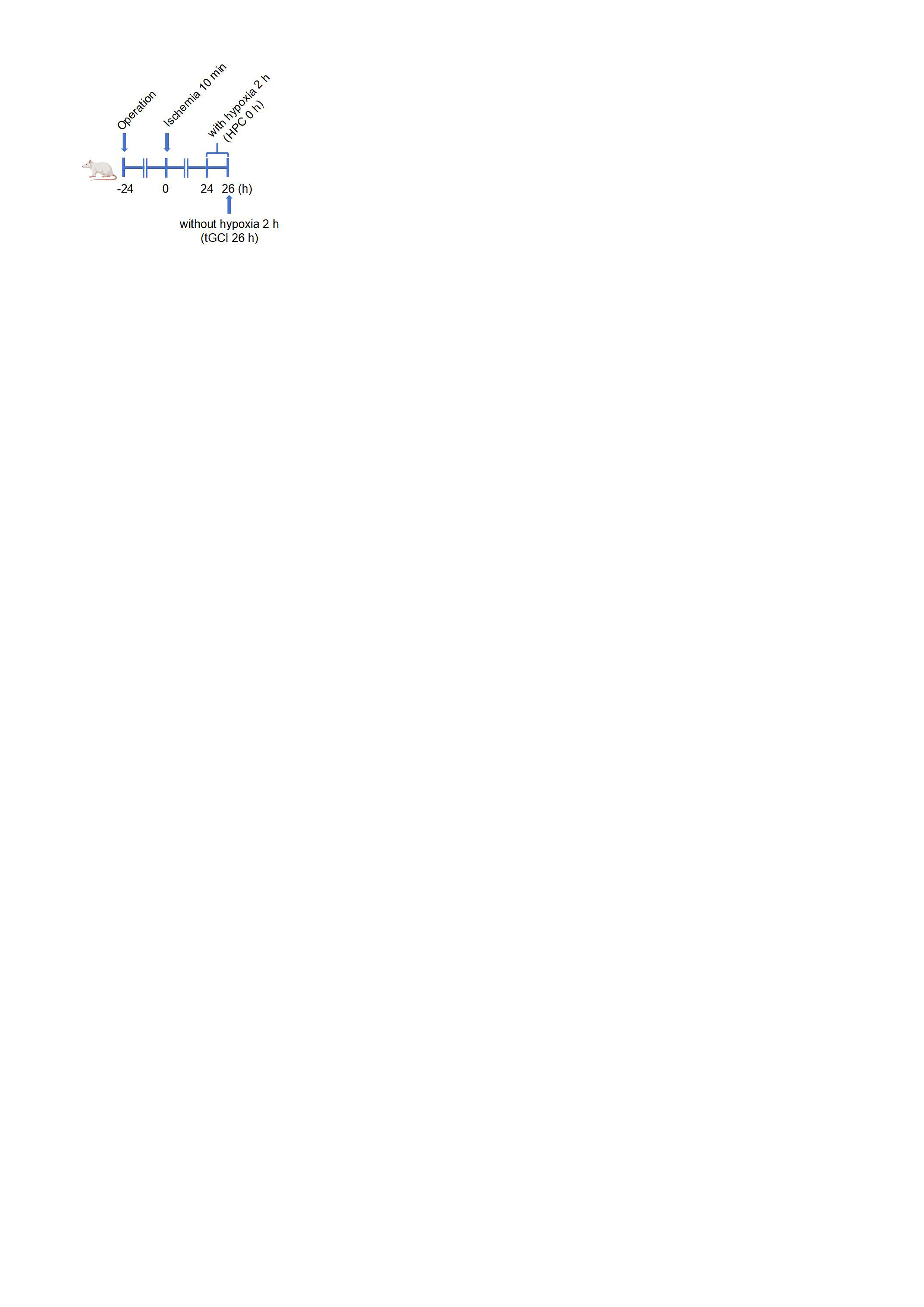

Supplement: Supplementary file 1 — Figure S1.Flow diagram for constructing tGCI and HPC models. HPC, hypoxic postconditioning; tGCI, transient global cerebral ischemia. Figure S2. Principal Components Analysis (PCA). Principal component analysis was performed on nine samples according to the transcripts per million counts. Each dot on the plot represents one sample. Blue, green and red indicate sham, tGCI and HPC groups, respectively. HPC, hypoxic postconditioning; sham, sham‐operated; tGCI, transient global cerebral ischemia. Figure S3. Target genes of 5 studied piRNAs. (A) Bar graph represents the number of target genes of top 5 differentially expressed piRNAs. (B–F) Visualization of top 5 differentially expressed piRNAs and their associated target genes with Cytoscape. piRNAs, piwi‐interacting RNAs. Figure S4. The prediction of piRNA transposon. (A) Distribution of transposon classified statistics chart. (B–F) The transposon percentages of top five differentially expressed piRNAs are summarized in the pie chart. piRNAs, piwi‐interacting RNAs. For the characteristics of predicted transposons, see Figure S5. Figure S5. Characteristics of predicted transposons. The tabulated and bar graph data show chromosomal location and predicted transposons number of top 5 piRNAs differentially expressed. Figure S6. Downregulation of rno_piR_011022 protects neuron against tGCI in CA1. (A) Representative microphotographs of cresyl violet staining, NeuN immunostaining and TUNEL staining in the hippocampus at 7 days after tGCI with sh‐Con or LV‐Con administration. sham+sh‐Con group (a1–a5), injection with sh‐Con without ischemia or hypoxia; sham+LV‐Con group (b1–b5), injection with LV‐Con without ischemia or hypoxia; tGCI+sh‐Con group (c1–c5), injection with sh‐Con before tGCI; tGCI+LV‐Con group (d1–d5), injection with LV‐Con before tGCI; HPC + sh‐Con group (e1–e5), injection with sh‐Con before HPC; HPC + LV‐Con group (f1–f5), injection with LV‐Con before HPC; Scale bar: a1–f1, a3–f3: 250 μm, a2–f2, a4–f4: 25 μm. (B [file CNS-31-e70295-s001.zip › cns70295-sup-0001-FigureS1.tiff]

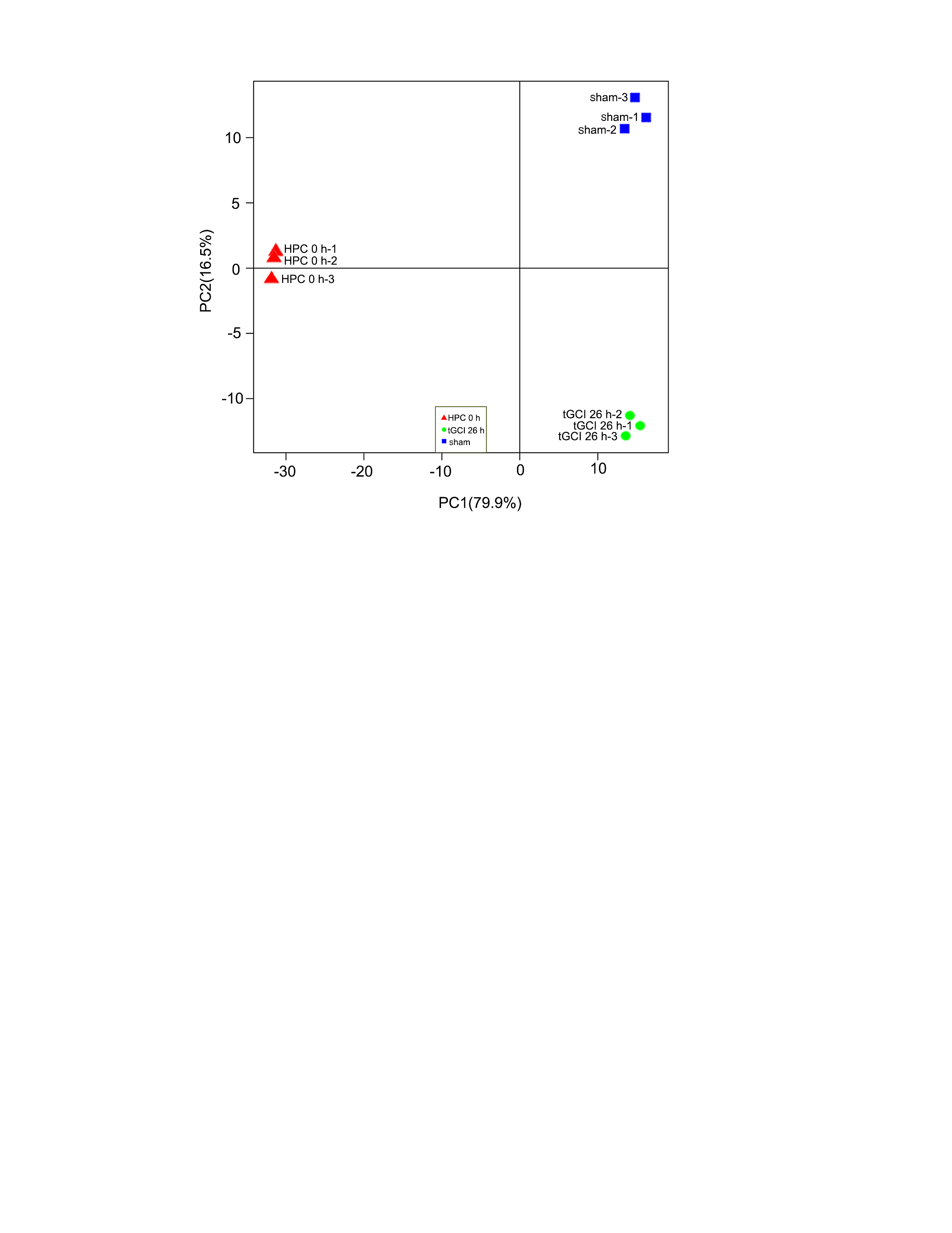

Supplement: Supplementary file 1 — Figure S1.Flow diagram for constructing tGCI and HPC models. HPC, hypoxic postconditioning; tGCI, transient global cerebral ischemia. Figure S2. Principal Components Analysis (PCA). Principal component analysis was performed on nine samples according to the transcripts per million counts. Each dot on the plot represents one sample. Blue, green and red indicate sham, tGCI and HPC groups, respectively. HPC, hypoxic postconditioning; sham, sham‐operated; tGCI, transient global cerebral ischemia. Figure S3. Target genes of 5 studied piRNAs. (A) Bar graph represents the number of target genes of top 5 differentially expressed piRNAs. (B–F) Visualization of top 5 differentially expressed piRNAs and their associated target genes with Cytoscape. piRNAs, piwi‐interacting RNAs. Figure S4. The prediction of piRNA transposon. (A) Distribution of transposon classified statistics chart. (B–F) The transposon percentages of top five differentially expressed piRNAs are summarized in the pie chart. piRNAs, piwi‐interacting RNAs. For the characteristics of predicted transposons, see Figure S5. Figure S5. Characteristics of predicted transposons. The tabulated and bar graph data show chromosomal location and predicted transposons number of top 5 piRNAs differentially expressed. Figure S6. Downregulation of rno_piR_011022 protects neuron against tGCI in CA1. (A) Representative microphotographs of cresyl violet staining, NeuN immunostaining and TUNEL staining in the hippocampus at 7 days after tGCI with sh‐Con or LV‐Con administration. sham+sh‐Con group (a1–a5), injection with sh‐Con without ischemia or hypoxia; sham+LV‐Con group (b1–b5), injection with LV‐Con without ischemia or hypoxia; tGCI+sh‐Con group (c1–c5), injection with sh‐Con before tGCI; tGCI+LV‐Con group (d1–d5), injection with LV‐Con before tGCI; HPC + sh‐Con group (e1–e5), injection with sh‐Con before HPC; HPC + LV‐Con group (f1–f5), injection with LV‐Con before HPC; Scale bar: a1–f1, a3–f3: 250 μm, a2–f2, a4–f4: 25 μm. (B [file CNS-31-e70295-s001.zip › cns70295-sup-0002-FigureS2.tiff]

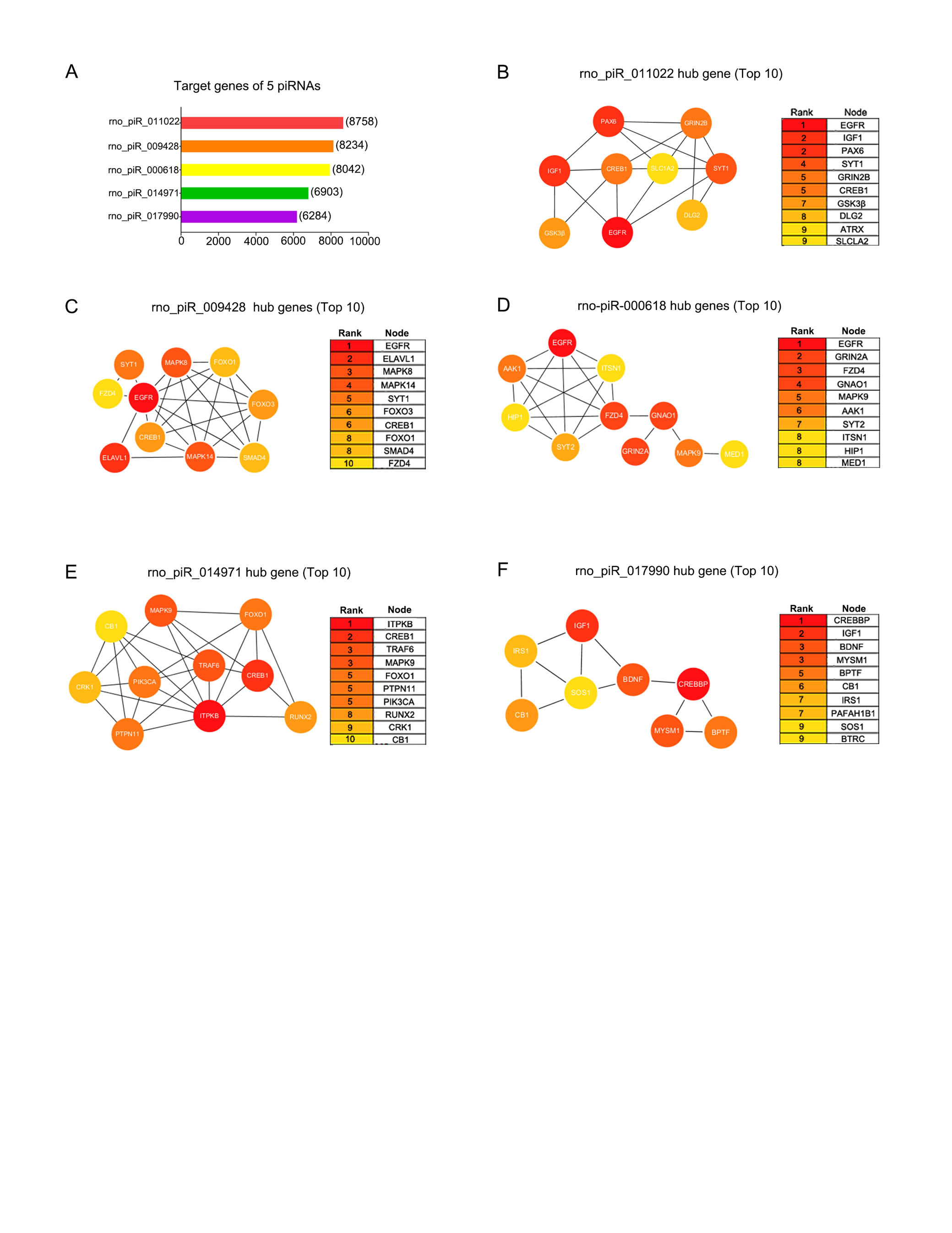

Supplement: Supplementary file 1 — Figure S1.Flow diagram for constructing tGCI and HPC models. HPC, hypoxic postconditioning; tGCI, transient global cerebral ischemia. Figure S2. Principal Components Analysis (PCA). Principal component analysis was performed on nine samples according to the transcripts per million counts. Each dot on the plot represents one sample. Blue, green and red indicate sham, tGCI and HPC groups, respectively. HPC, hypoxic postconditioning; sham, sham‐operated; tGCI, transient global cerebral ischemia. Figure S3. Target genes of 5 studied piRNAs. (A) Bar graph represents the number of target genes of top 5 differentially expressed piRNAs. (B–F) Visualization of top 5 differentially expressed piRNAs and their associated target genes with Cytoscape. piRNAs, piwi‐interacting RNAs. Figure S4. The prediction of piRNA transposon. (A) Distribution of transposon classified statistics chart. (B–F) The transposon percentages of top five differentially expressed piRNAs are summarized in the pie chart. piRNAs, piwi‐interacting RNAs. For the characteristics of predicted transposons, see Figure S5. Figure S5. Characteristics of predicted transposons. The tabulated and bar graph data show chromosomal location and predicted transposons number of top 5 piRNAs differentially expressed. Figure S6. Downregulation of rno_piR_011022 protects neuron against tGCI in CA1. (A) Representative microphotographs of cresyl violet staining, NeuN immunostaining and TUNEL staining in the hippocampus at 7 days after tGCI with sh‐Con or LV‐Con administration. sham+sh‐Con group (a1–a5), injection with sh‐Con without ischemia or hypoxia; sham+LV‐Con group (b1–b5), injection with LV‐Con without ischemia or hypoxia; tGCI+sh‐Con group (c1–c5), injection with sh‐Con before tGCI; tGCI+LV‐Con group (d1–d5), injection with LV‐Con before tGCI; HPC + sh‐Con group (e1–e5), injection with sh‐Con before HPC; HPC + LV‐Con group (f1–f5), injection with LV‐Con before HPC; Scale bar: a1–f1, a3–f3: 250 μm, a2–f2, a4–f4: 25 μm. (B [file CNS-31-e70295-s001.zip › cns70295-sup-0003-FigureS3.tif]

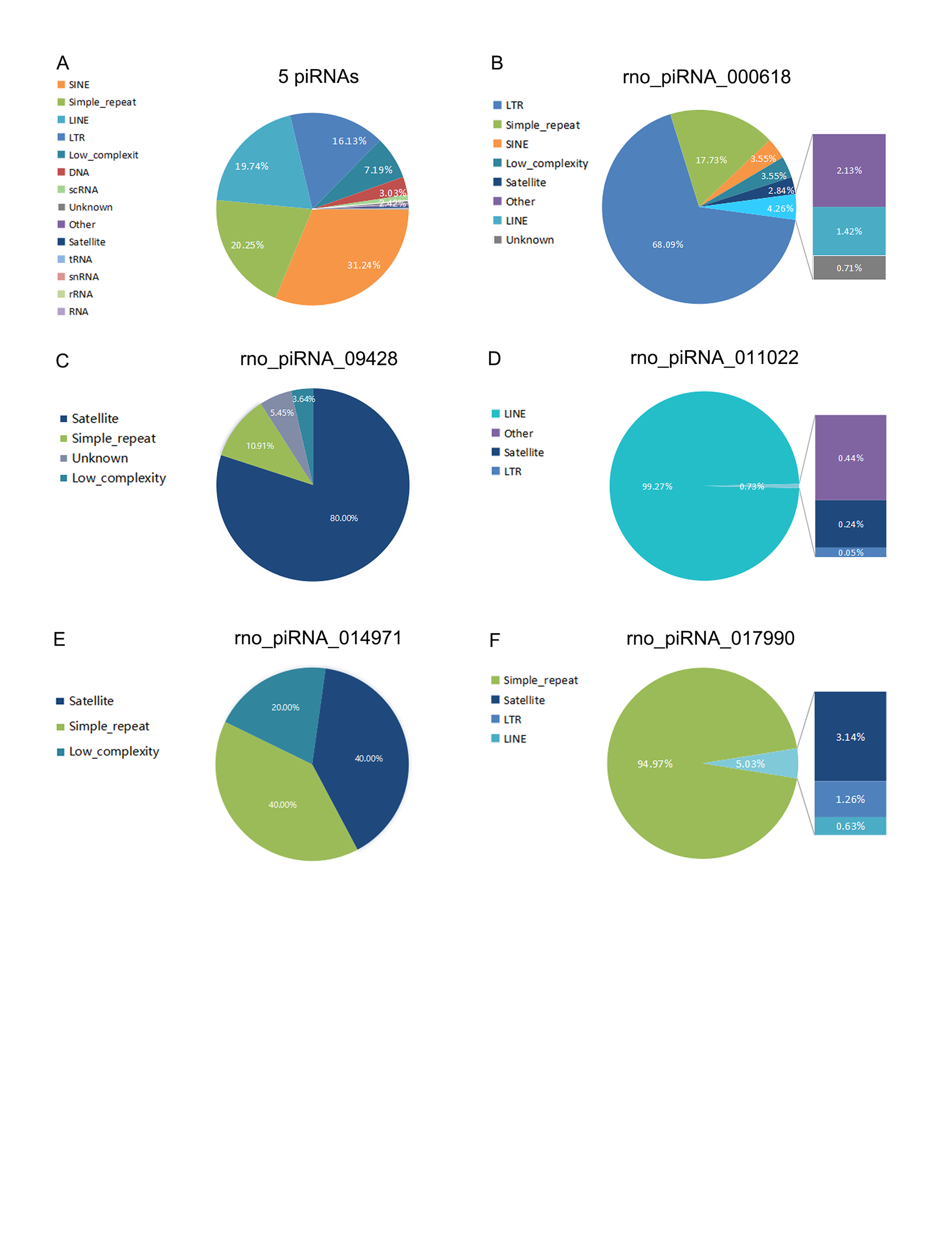

Supplement: Supplementary file 1 — Figure S1.Flow diagram for constructing tGCI and HPC models. HPC, hypoxic postconditioning; tGCI, transient global cerebral ischemia. Figure S2. Principal Components Analysis (PCA). Principal component analysis was performed on nine samples according to the transcripts per million counts. Each dot on the plot represents one sample. Blue, green and red indicate sham, tGCI and HPC groups, respectively. HPC, hypoxic postconditioning; sham, sham‐operated; tGCI, transient global cerebral ischemia. Figure S3. Target genes of 5 studied piRNAs. (A) Bar graph represents the number of target genes of top 5 differentially expressed piRNAs. (B–F) Visualization of top 5 differentially expressed piRNAs and their associated target genes with Cytoscape. piRNAs, piwi‐interacting RNAs. Figure S4. The prediction of piRNA transposon. (A) Distribution of transposon classified statistics chart. (B–F) The transposon percentages of top five differentially expressed piRNAs are summarized in the pie chart. piRNAs, piwi‐interacting RNAs. For the characteristics of predicted transposons, see Figure S5. Figure S5. Characteristics of predicted transposons. The tabulated and bar graph data show chromosomal location and predicted transposons number of top 5 piRNAs differentially expressed. Figure S6. Downregulation of rno_piR_011022 protects neuron against tGCI in CA1. (A) Representative microphotographs of cresyl violet staining, NeuN immunostaining and TUNEL staining in the hippocampus at 7 days after tGCI with sh‐Con or LV‐Con administration. sham+sh‐Con group (a1–a5), injection with sh‐Con without ischemia or hypoxia; sham+LV‐Con group (b1–b5), injection with LV‐Con without ischemia or hypoxia; tGCI+sh‐Con group (c1–c5), injection with sh‐Con before tGCI; tGCI+LV‐Con group (d1–d5), injection with LV‐Con before tGCI; HPC + sh‐Con group (e1–e5), injection with sh‐Con before HPC; HPC + LV‐Con group (f1–f5), injection with LV‐Con before HPC; Scale bar: a1–f1, a3–f3: 250 μm, a2–f2, a4–f4: 25 μm. (B [file CNS-31-e70295-s001.zip › cns70295-sup-0004-FigureS4.tiff]

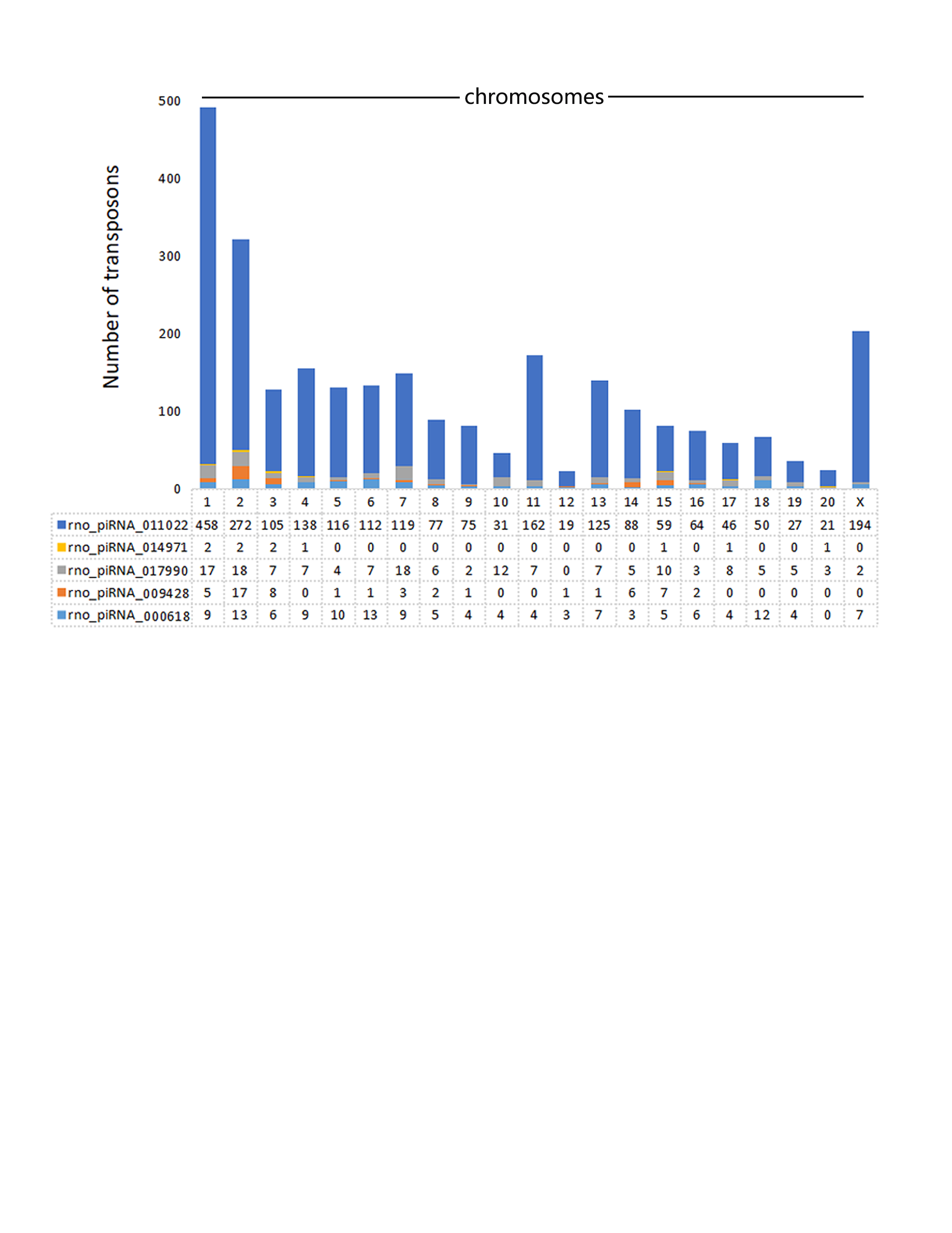

Supplement: Supplementary file 1 — Figure S1.Flow diagram for constructing tGCI and HPC models. HPC, hypoxic postconditioning; tGCI, transient global cerebral ischemia. Figure S2. Principal Components Analysis (PCA). Principal component analysis was performed on nine samples according to the transcripts per million counts. Each dot on the plot represents one sample. Blue, green and red indicate sham, tGCI and HPC groups, respectively. HPC, hypoxic postconditioning; sham, sham‐operated; tGCI, transient global cerebral ischemia. Figure S3. Target genes of 5 studied piRNAs. (A) Bar graph represents the number of target genes of top 5 differentially expressed piRNAs. (B–F) Visualization of top 5 differentially expressed piRNAs and their associated target genes with Cytoscape. piRNAs, piwi‐interacting RNAs. Figure S4. The prediction of piRNA transposon. (A) Distribution of transposon classified statistics chart. (B–F) The transposon percentages of top five differentially expressed piRNAs are summarized in the pie chart. piRNAs, piwi‐interacting RNAs. For the characteristics of predicted transposons, see Figure S5. Figure S5. Characteristics of predicted transposons. The tabulated and bar graph data show chromosomal location and predicted transposons number of top 5 piRNAs differentially expressed. Figure S6. Downregulation of rno_piR_011022 protects neuron against tGCI in CA1. (A) Representative microphotographs of cresyl violet staining, NeuN immunostaining and TUNEL staining in the hippocampus at 7 days after tGCI with sh‐Con or LV‐Con administration. sham+sh‐Con group (a1–a5), injection with sh‐Con without ischemia or hypoxia; sham+LV‐Con group (b1–b5), injection with LV‐Con without ischemia or hypoxia; tGCI+sh‐Con group (c1–c5), injection with sh‐Con before tGCI; tGCI+LV‐Con group (d1–d5), injection with LV‐Con before tGCI; HPC + sh‐Con group (e1–e5), injection with sh‐Con before HPC; HPC + LV‐Con group (f1–f5), injection with LV‐Con before HPC; Scale bar: a1–f1, a3–f3: 250 μm, a2–f2, a4–f4: 25 μm. (B [file CNS-31-e70295-s001.zip › cns70295-sup-0005-FigureS5.tiff]

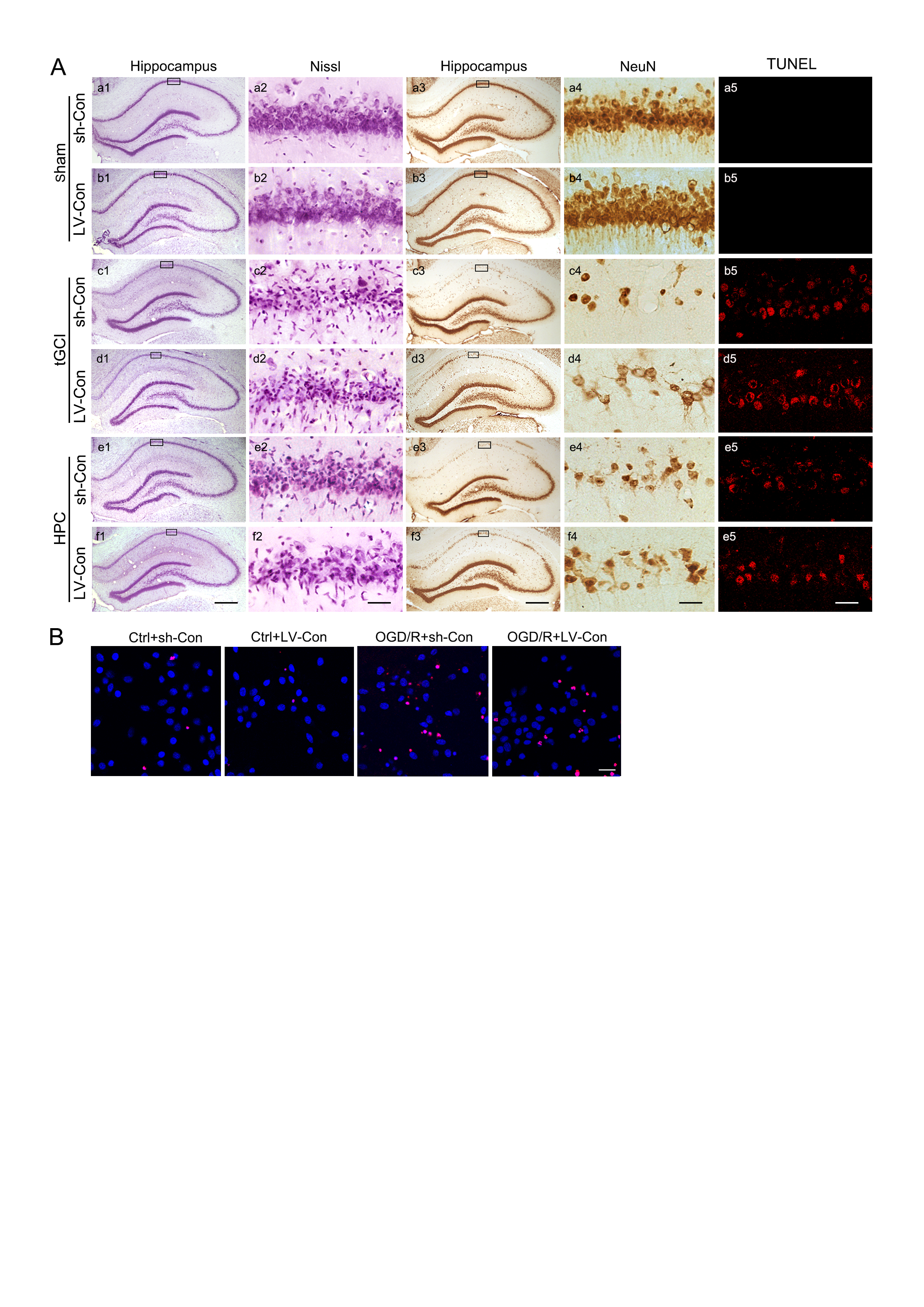

Supplement: Supplementary file 1 — Figure S1.Flow diagram for constructing tGCI and HPC models. HPC, hypoxic postconditioning; tGCI, transient global cerebral ischemia. Figure S2. Principal Components Analysis (PCA). Principal component analysis was performed on nine samples according to the transcripts per million counts. Each dot on the plot represents one sample. Blue, green and red indicate sham, tGCI and HPC groups, respectively. HPC, hypoxic postconditioning; sham, sham‐operated; tGCI, transient global cerebral ischemia. Figure S3. Target genes of 5 studied piRNAs. (A) Bar graph represents the number of target genes of top 5 differentially expressed piRNAs. (B–F) Visualization of top 5 differentially expressed piRNAs and their associated target genes with Cytoscape. piRNAs, piwi‐interacting RNAs. Figure S4. The prediction of piRNA transposon. (A) Distribution of transposon classified statistics chart. (B–F) The transposon percentages of top five differentially expressed piRNAs are summarized in the pie chart. piRNAs, piwi‐interacting RNAs. For the characteristics of predicted transposons, see Figure S5. Figure S5. Characteristics of predicted transposons. The tabulated and bar graph data show chromosomal location and predicted transposons number of top 5 piRNAs differentially expressed. Figure S6. Downregulation of rno_piR_011022 protects neuron against tGCI in CA1. (A) Representative microphotographs of cresyl violet staining, NeuN immunostaining and TUNEL staining in the hippocampus at 7 days after tGCI with sh‐Con or LV‐Con administration. sham+sh‐Con group (a1–a5), injection with sh‐Con without ischemia or hypoxia; sham+LV‐Con group (b1–b5), injection with LV‐Con without ischemia or hypoxia; tGCI+sh‐Con group (c1–c5), injection with sh‐Con before tGCI; tGCI+LV‐Con group (d1–d5), injection with LV‐Con before tGCI; HPC + sh‐Con group (e1–e5), injection with sh‐Con before HPC; HPC + LV‐Con group (f1–f5), injection with LV‐Con before HPC; Scale bar: a1–f1, a3–f3: 250 μm, a2–f2, a4–f4: 25 μm. (B [file CNS-31-e70295-s001.zip › cns70295-sup-0006-FigureS6.tiff]

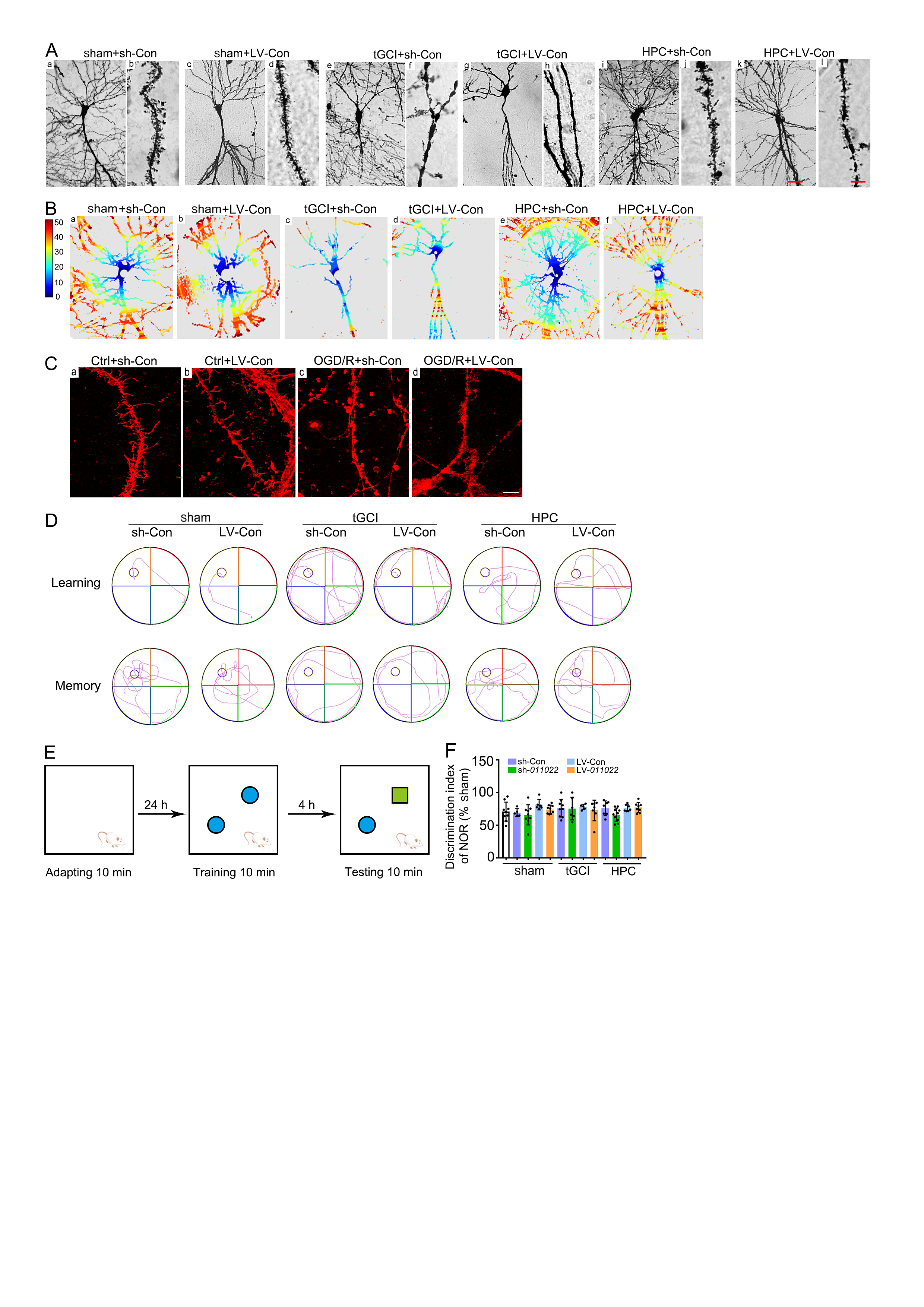

Supplement: Supplementary file 1 — Figure S1.Flow diagram for constructing tGCI and HPC models. HPC, hypoxic postconditioning; tGCI, transient global cerebral ischemia. Figure S2. Principal Components Analysis (PCA). Principal component analysis was performed on nine samples according to the transcripts per million counts. Each dot on the plot represents one sample. Blue, green and red indicate sham, tGCI and HPC groups, respectively. HPC, hypoxic postconditioning; sham, sham‐operated; tGCI, transient global cerebral ischemia. Figure S3. Target genes of 5 studied piRNAs. (A) Bar graph represents the number of target genes of top 5 differentially expressed piRNAs. (B–F) Visualization of top 5 differentially expressed piRNAs and their associated target genes with Cytoscape. piRNAs, piwi‐interacting RNAs. Figure S4. The prediction of piRNA transposon. (A) Distribution of transposon classified statistics chart. (B–F) The transposon percentages of top five differentially expressed piRNAs are summarized in the pie chart. piRNAs, piwi‐interacting RNAs. For the characteristics of predicted transposons, see Figure S5. Figure S5. Characteristics of predicted transposons. The tabulated and bar graph data show chromosomal location and predicted transposons number of top 5 piRNAs differentially expressed. Figure S6. Downregulation of rno_piR_011022 protects neuron against tGCI in CA1. (A) Representative microphotographs of cresyl violet staining, NeuN immunostaining and TUNEL staining in the hippocampus at 7 days after tGCI with sh‐Con or LV‐Con administration. sham+sh‐Con group (a1–a5), injection with sh‐Con without ischemia or hypoxia; sham+LV‐Con group (b1–b5), injection with LV‐Con without ischemia or hypoxia; tGCI+sh‐Con group (c1–c5), injection with sh‐Con before tGCI; tGCI+LV‐Con group (d1–d5), injection with LV‐Con before tGCI; HPC + sh‐Con group (e1–e5), injection with sh‐Con before HPC; HPC + LV‐Con group (f1–f5), injection with LV‐Con before HPC; Scale bar: a1–f1, a3–f3: 250 μm, a2–f2, a4–f4: 25 μm. (B [file CNS-31-e70295-s001.zip › cns70295-sup-0007-FigureS7.tiff]
